# A *PECTIN METHYLESTERASE* gene at the maize *Ga1* locus confers male function in unilateral cross-incompatibility

**DOI:** 10.1038/s41467-018-06139-8

**Published:** 2018-09-10

**Authors:** Zhaogui Zhang, Baocai Zhang, Zhibin Chen, Dongmei Zhang, Huairen Zhang, Hang Wang, Yu’e Zhang, Darun Cai, Juan Liu, Senlin Xiao, Yanqing Huo, Jie Liu, Lanjun Zhang, Mingming Wang, Xu Liu, Yongbiao Xue, Li Zhao, Yihua Zhou, Huabang Chen

**Affiliations:** 1 State Key Laboratory of Plant Cell and Chromosome Engineering, Institute of Genetics and Developmental Biology, Chinese Academy of Sciences, 100101 Beijing, China; 2 University of Chinese Academy of Sciences, 100039 Beijing, China; 3 State Key Laboratory of Plant Genomics, Institute of Genetics and Developmental Biology, Chinese Academy of Sciences, 100101 Beijing, China; 4 Institute of Cotton Research, Chinese Academy of Agricultural Sciences, 455000 Anyang, China

## Abstract

Unilateral cross-incompatibility (UCI) is a unidirectional inter/intra-population reproductive barrier when both parents are self-compatible. Maize *Gametophyte factor1* (*Ga1*) is an intraspecific UCI system and has been utilized in breeding. However, the mechanism underlying maize UCI specificity has remained mysterious for decades. Here, we report the cloning of *ZmGa1P*, a pollen-expressed *PECTIN METHYLESTERASE* (*PME*) gene at the *Ga1* locus that can confer the male function in the maize UCI system. Homozygous transgenic plants expressing *ZmGa1P* in a *ga1* background can fertilize *Ga1-S* plants and can be fertilized by pollen of *ga1* plants. ZmGa1P protein is predominantly localized to the apex of growing pollen tubes and may interact with another pollen-specific PME protein, ZmPME10-1, to maintain the state of pectin methylesterification required for pollen tube growth in *Ga1-S* silks. Our study discloses a PME-mediated UCI mechanism and provides a tool to manipulate hybrid breeding.

## Abstract (web summary)

Unilateral cross-incompatibility between certain varieties of maize prevents cross-fertilization and can facilitate hybrid breeding. Here the authors show that a *PECTIN METHYLESTERASE* gene is able to overcome this reproductive barrier and confer fertility when expressed in pollen of the male parent.

## Introduction

Maize is a versatile crop grown worldwide and is used as human food, animal feed, and industrial raw materials for starch and biofuel production. World-wide corn production and consumption exceeded 38 billion bushels in 2016 and is continually increasing (2016 data, see ref. ^1^), benefiting from an increased maize yield that has largely resulted from harnessing heterosis as well as improved agronomic practice. To optimize heterosis in hybrid seed production, avoiding undesired pollen is required, which is also important for organic and specialty maize productions. Multiple efforts, including temporal and spatial isolations, have been made to exclude undesired pollen; however, the technique remains challenging given that maize is a cross-pollinating crop with a large volume of pollen that can travel long distances via wind and insects^2,3^. Unilateral cross incompatibility (UCI) may offer an effective method to manipulate pollen flow in commercial maize production^4–6^.

UCI is a postpollination reproductive barrier that unidirectionally prevents hybridization when male and female parents are self-compatible^4,5^. Since the early 1900s, a non-Mendelian segregation ratio at the *sugary 1* locus has been noted in crosses between popcorn and sweet corns^7^, resulting from UCI controlled by one linked *Gametophyte factor1* (*Ga1*) locus in popcorn^2,4,8–10^. Genetic analyses revealed that maize UCI is governed by male-expressed and female-expressed components within the *Ga1* locus^5,9–11^, generally resembling *S*-locus structure, a complex locus that confers self-incompatibility (SI) in numerous plant species^12–14^. However, in contrast to SI, which requires a suite of polymorphic haplotypes to distinguish individuals within a population, the *Ga1* system is composed of three different haplotypes: the *Ga1-S* haplotype possesses both male and female determinants, the *Ga1-M* haplotype only harbors the male determinant, and the *ga1* haplotype (wild type) lacks both. During fertilization, *Ga1-S* plants completely reject the pollen of *ga1* due to the female barrier but accept pollen of *Ga1-S* and *Ga1-M* haplotypes, though the selfing and reciprocal crosses are compatible^2,3^. Many efforts have been made to identify the male and female determinants^3,11,15^. Recently, *ZmPME3* in the *Ga1* region was found to be specifically expressed in the silks of *Ga1-S* plants, serving as a likely candidate for the female function^15^. However, the mechanism underlying the male function in maize UCI remains elusive.

In this study, we clone a male determinant *ZmGa1P* at the *Ga1* locus and reveal that expression of this pollen-specific *PME* can overcome the barrier in the *Ga1-S* silks. The interaction of ZmGa1P and another pollen-specific PME, ZmPME10-1, may form a protein complex to maintain the equilibrium of the apical cell wall dynamics during pollen tube growth, offering a mechanistic view for maize UCI.

## Results

### Genetic cloning of a male determinant at the *Ga1* locus

**Fig. 1:**
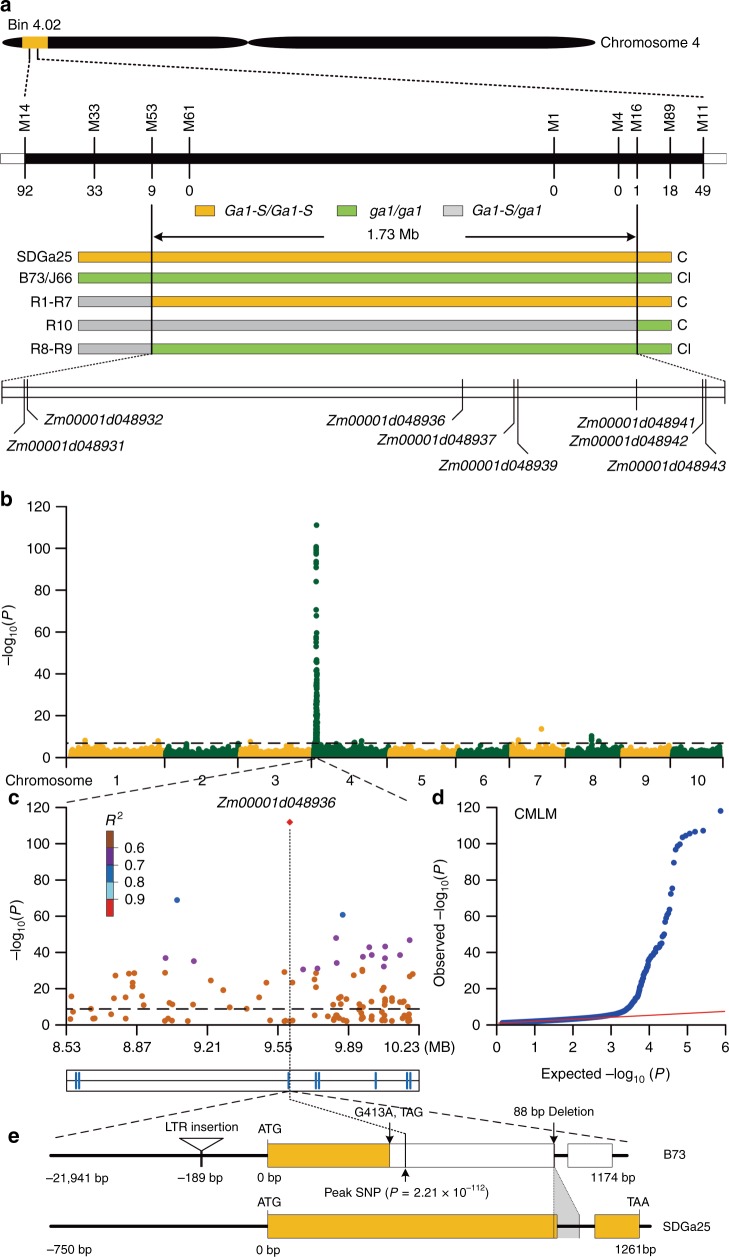
Map-based cloning of the pollen determinant. **a** Physical mapping, showing Bin 4.02 narrowed to a region between markers M53 and M16 contains 8 annotated genes. Recombinants are indicated below the markers. R1-R10, recombinants. C, cross-compatible; CI, cross-incompatible. **b** Genome-wide association analysis of the pollen determinant. The horizontal dashed line depicts the Bonferroni-adjusted significance threshold (*P* = 2.15 × 10^−8^). **c** The genome-wide association signals in the 8.53–10.23 Mb interval. The peak SNP is positioned by a vertical dashed line with a red diamond. The eight annotated genes are indicated by the blue bars at the bottom. **d** Quantile–quantile plot under a compressed mixed linear model. **e** Gene structure of *Zm00001d048936* in B73 (*ga1*) and SDGa25 (*Ga1-S*)

Maize *Ga1* was reported in the 1900s but has remained an open issue for a century^4^. To address the underlying mechanism of *Ga1*, we utilized a map-based cloning strategy to isolate male determinant gene. In this study, approximately 21,600 individuals from two segregating populations were subjected to recombinant screening. The region containing male determinant gene was narrowed to a 1.73-Mb interval between markers M53 and M16 (Supplementary Table 1), in which eight protein-coding genes were annotated based on the *B73 AGP v4* sequence^16^ (Fig. 1a). To identify male determinant gene, 946 inbred lines of *Ga1-S*, *Ga1-M* and *ga1* haplotypes were genotyped using a 600-K SNP chip for genome-wide association analysis (Fig. 1b–d). Using a compressed mixed linear model that incorporates population structures as fixed effects and kinships as random effects to evaluate the *P* values (Supplementary Fig. 1), 167 of 174 male determinant associated SNPs (*P* < 2.15 × 10^−8^) were found to be intensively distributed in the mapping interval (Supplementary Table 2), and the peak SNP (*P* = 2.21 × 10^−112^) was located within *Zm00001d048936* (Fig. 1b–d), suggesting this gene as the most likely candidate.

We then constructed a bacterial artificial chromosome (BAC) library of SDGa25 (*Ga1-S*) to investigate the gene structure of *Zm00001d048936*. A 135-kb BAC clone containing two copies of *Zm00001d048936* was identified. In this BAC, copy_1 is intact and expressed, but copy_2 is interrupted by a long-terminal-repeat retrotransposon (LTR) and not transcribed (Supplementary Fig. 2). In addition, 86% of the remaining BAC sequences belong to several types of transposons (Supplementary Data 1), resulting in weak collinearity between the 135-kb BAC and the B73 (*ga1*) reference sequence. However, alignment of the intact copy of *Zm00001d048936* with B73 revealed 26 hits in the mapping region. Only one hit, the *Zm00001d048936* gene in B73, exhibits a gene structure identical to that in SDGa25, whereas the other 25 hits are partial fragments (Supplementary Figs. 3). The *Zm00001d048936* sequence in SDGa25 and B73 was further compared to determine genomic variations between *Ga1-S* and *ga1* lines. In addition to synonymous and missense variations (Supplementary Figs. 4, 5), B73 has a 21,254-bp LTR insertion at the promoter region (−189-bp), an 88-bp deletion (at the junction of the 1st exon and intron), and a premature nonsense point mutation (G413A) (Fig. 1e). To investigate whether these variations are widespread in different maize lines, the 88-bp deletion was used to genotype the above 946 inbred lines. This deletion was consistently present in all *ga1* lines, but absent in *Ga1-S* and *Ga1-M* lines (Supplementary Fig. 6).

**Fig. 2:**
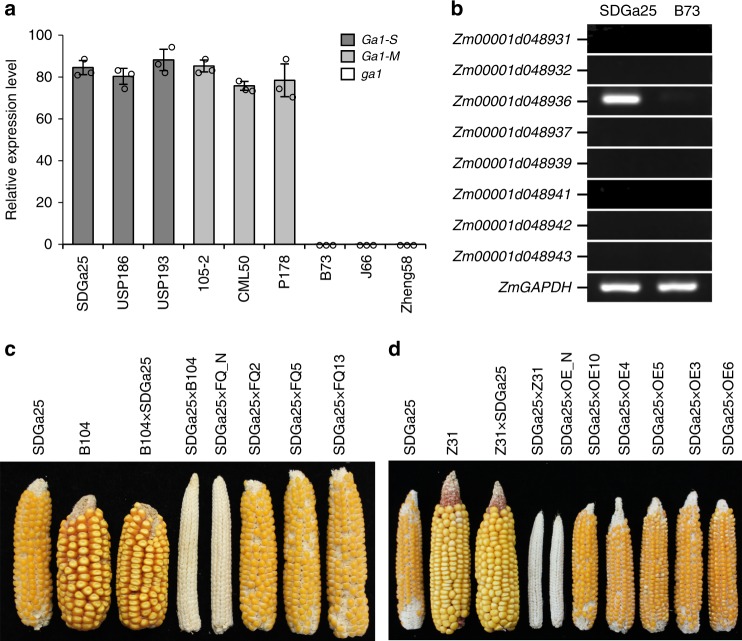
Validation of *Zm00001d048936* as a pollen determinant. **a** Quantitative expression analysis of *Zm0001d048936* in pollen of *Ga1-S*, *Ga1-M*, and *ga1* lines. Error bars represent the mean ± SD (*n* = 3). **b** Examination of the eight candidate gene expression in pollen of SDGa25 (*Ga1-S*) and B73 (*ga1*). *ZmGAPDH was* used as an internal control. **c**, **d** Cross-compatibility analyses, showing ears of self-pollinations and reciprocal crosses between the SDGa25 (*Ga1-S*) and B104 or Z31 (*ga1*, control plants). The SDGa25 ears pollinated with non-transgenic controls (FQ_N and OE_N) and transgenic plants expressing *ZmGa1P*. **c** Transgenic plants made with the construct shown in Supplementary Fig 7a, **d** T_2_ generation of transgenic plants made with the construct shown in Supplementary Fig 8a

Genomic variations may disturb gene structure and/or gene expression. The transcripts of *Zm00001d048936* were detected exclusively in pollen of *Ga1-S* and *Ga1-M* lines, and not in that of *ga1* lines (Fig. 2a). *Zm00001d048936* is also the only gene expressed in SDGa25 pollen among the eight candidates located within the mapping region (Fig. 2b). All these data demonstrated that *Zm00001d048936* is a candidate for the male function.

### Expressing *Zm00001d048936* endows *ga1* with male function

To verify *Zm00001d048936* as a male determinant, a 6,287-bp genomic fragment of *Zm00001d048936* cloned from SDGa25 was introduced into the inbred line B104 (*ga1*) (Supplementary Fig. 7a). Pollen from three independent *T*_1_ transgenic (FQ2, FQ5, and FQ13) and nontransgenic plants were used to fertilize detasseled SDGa25 (*Ga1-S*) plants, separately. All the SDGa25 plants fertilized with pollen from the transgenic plants exhibited a full seed-set (Fig. 2c), but those fertilized with pollen from nontransgenic B104 had no seeds, providing genetic evidence for the conclusion that *Zm00001d048936* is a pollen determinant. In addition, a construct harboring full-length cDNA of *Zm00001d048936* from SDGa25 (Supplementary Fig. 8a) was introduced into maize inbred line Z31 (*ga1*) to generate over-expression plants (OE). We thereby pollinated detasseled SDGa25 plants with pollen from Z31 and five independent transgenic lines (OE3, OE4, OE5, OE6, and OE10) at *T*_0_, *T*_1_, and *T*_2_ generations, separately. A full seed-set was observed using transgenic pollen, but no seed-set was obtained using pollen from Z31 (Fig. 2d and Supplementary Fig. 8b). PCR analyses further confirmed the heterozygotic genotype in the seeds resulting from cross-pollination (Supplementary Figs. 7b and 8c, d) and corroborated the expression of *Zm00001d048936* in the pollen of plants expressing the genomic fragment and of the OE lines (Supplementary Figs. 7c, 8e). Therefore, expressing *Ga1*-type *Zm00001d048936* endows *ga1* pollen with the ability to penetrate the female barrier of *Ga1-S*.

To determine whether *Zm00001d048936* has female function, the five *T*_2_ OE lines that exhibited high level of *Zm00001d048936* transcripts in silks were pollinated with B73 (*ga1*) pollen. The full seed-set suggested that *Zm00001d048936* does not possess the function of the female barrier (Supplementary Fig. 9). We thus conclude that *Zm00001d048936*, which is designated *ZmGa1P*, is a pollen/male determinant of the *Ga1* locus.

### ZmGa1P encodes a pollen PME and modulates pollen tube growth

Alignment of the *ZmGa1P* sequence revealed that this gene encodes a putative PME, a class of critical factors affecting pollen tube growth^17^. Fertilization in plants involves a series of physiological processes, including hydration and germination of pollen grains, growth of pollen tubes for penetration and navigation in the transmitting tract to deliver sperm, and recognition between sperm nuclei and egg cells^18^. To dissect the cause of UCI regulated by *Ga1*, we analyzed pollen tube growth in silks. Although both pollen types can germinate on *Ga1-S* stigma, growth of *ga1* pollen tubes was significantly inhibited compared with *Ga1-S* (Supplementary Fig. 10a, b). Our results, together with the results from other groups^3,11,19^, revealed that the cross-incompatibility of the *Ga1* locus is caused by abnormal pollen tube growth.

**Fig. 3:**
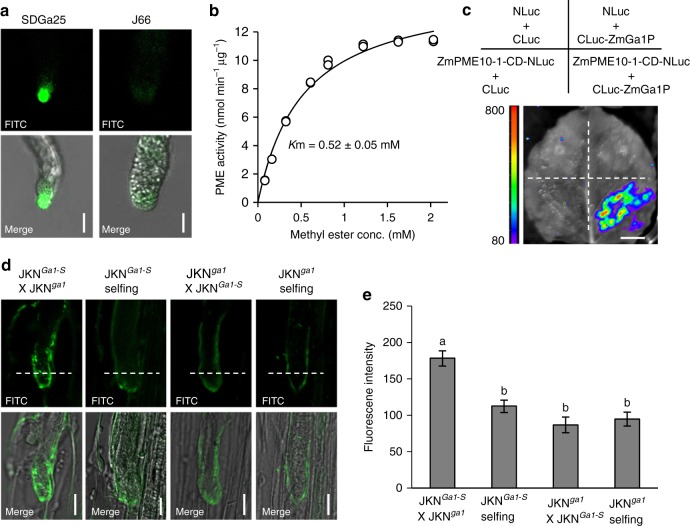
Characterization of ZmGa1P biochemical properties. **a** Immuno-staining the pollen tubes of SDGa25 (*Ga1-S*) and J66 (*ga1*) using the anti-ZmGa1P antibody. Scale bar = 10 μm. **b** Kinetic analysis of PME activity of secretory proteins in the fraction 8 of SDGa25. Error bars represent the mean ± SD of three replicates. **c** Split-luciferase complementation assay showing the interaction between ZmGa1P and the PME domain (CD) of ZmPME10-1 in *N. benthamiana* leaves infiltrated with the construct combinations shown above. Scale bar = 1 cm. **d** Immuno-detecting highly methylesterified pectins in the longitudinally sectioned pollen tubes of near isogenic lines with LM20 antibody. Scale bars = 10 μm. FITC, Fluorescein isothiocyanate. **e** Quantification of the fluorescent signal intensity of immune-examinations, to show the varied pectin DM on *ga1* pollen tube walls growing in *Ga1-S* silks. Error bars represent the mean ± SE (*n* > 15 pollen tubes). a and b indicate that the means differ according to analysis of variance and Tukey’s test (*P* < 0.01)

Among multiple factors that affect pollen tube growth, the mechanical strength and plasticity of the pollen tube wall controlled by the equilibrium of pectin esterification-deesterification at the apical region of pollen tubes is crucial^20,21^. *ZmGa1P* was therefore proposed to function in pollen tubes and affect their wall properties. To verify this hypothesis, we examined *ZmGa1P* expression in various tissues of *Ga1-S* and *ga1* lines. Quantitative PCR analysis revealed that *ZmGa1P* is specifically expressed in *Ga1-S* pollen (Supplementary Fig. 10c). The encoded proteins were further found to be mainly localized at the pollen tube apex of *Ga1-S* plants, as revealed by immunolabeling the germinated pollen tubes with a specific anti-peptide antibody (Fig. 3a and Supplementary Fig. 10d, e). These findings are consistent with the view that PMEs function at the pollen tube apex^20^. Therefore, ZmGa1P is a PME candidate that specifically functions in the apex region of pollen tubes.

### Identifying ZmGa1P and ZmPME10-1 in *Ga1-S* pollen secretome

PME often functions in the apoplast^20,21^. To determine the biochemical property of ZmGa1P, we fractionated proteins secreted by pollen during germination. Comparing the fractionation profile of secretory proteins between *Ga1-S* and *ga1* pollen, an ~42-kD band was clearly detected in fractions 8–10 of *Ga1-S* (Supplementary Fig. 11a) but absent in *ga1* (Supplementary Fig. 11b). This band was recognized as ZmGa1P by the anti-ZmGa1P antibody (Supplementary Fig. 11a), indicating that *Ga1-S* pollen secreted large amounts of ZmGa1P. The pectoplate assay further revealed obvious PME activity in fractions 6–10 (Supplementary Fig. 11), suggesting that these fractions likely include active PMEs. We then subjected fractions 6–10 of *Ga1-S* and *ga1* pollen to LC-MS/MS analyses. Secretome analysis identified approximately one hundred proteins in each fraction (Supplementary Data 2). ZmGa1P and Zm00001d026422 (designated as ZmPME10-1) were two major annotated PMEs in *Ga1-S* pollen, whereas ZmPME10-1 was the most abundant PME in *ga1* pollen (Supplementary Figs. 12, 13 and Supplementary Table 3). Therefore, ZmGa1P plus ZmPME10-1 and ZmPME10-1 appeared to be the dominant PMEs in *Ga1-S* and *ga1* pollen, respectively. We selected fraction 8 as a representative to analyze PME activity. The fraction of *Ga1-S* line exhibited saturable kinetics with a *K*m of 0.52 mM (Fig. 3b); the value was 0.44 mM in *ga1* lines (Supplementary Fig. 14). Hence, the PME activity in *Ga1-S* and *ga1* lines may mainly result from the presence of ZmGa1P plus ZmPME10-1 and ZmPME10-1 in the pollen tubes, respectively.

### A PME complex controls pectin esterification of pollen tubes

Although the detected PMEs in *Ga1-S* and *ga1* pollen tubes exhibited obvious PME activity in vitro, only the pollen harboring ZmGa1P can penetrate the silk barrier of the *Ga1* locus, suggesting that the *in planta* PME activity mediated by ZmGa1P may differ from that in *ga1* lines. The presence of ZmGa1P and ZmPME10-1 together in the *Ga1-S* line prompted us to hypothesize that the two proteins may interact. We treated the secretory proteins of fraction 8 with disuccinimidyl suberate (DSS), a widely used chemical for protein cross-linking analysis. The 42-kD ZmGa1P shifted to a higher molecular weight ~100 kD (Supplementary Fig. 15a), indicating the existence of a protein complex. By subjecting the ~100-kD cross-linked band to LC-MS/MS analysis, ZmGa1P and ZmPME10-1 were identified as the most likely components in this complex (Supplementary Fig. 15b and Supplementary Data 3). To obtain *in planta* evidence, we crosslinked the protein extracts from unpollinated and pollinated *Ga1-S* silks using DSS. The ~100-kD protein complex was also found in the self-pollinated *Ga1-S* silks (Supplementary Fig. 15c). Application of the split-luciferase complementary assay in the transiently transformed tobacco leaves further corroborated the interaction between ZmGa1P and the PME domain or signal peptide-depleted version of ZmPME10-1 (Fig. 3c and Supplementary Fig. 15d).

PMEs catalyze pectin deesterification^20^. To visualize the in vivo difference in the degree of methyl esterification (DM) at the pollen-tube apexes, we developed near isogenic lines JKN^*Ga1-S*^ and JKN^*ga1*^ that differ at the *Ga1* locus and compared the DM of the pollen tube walls between selfing and reciprocal crosses. The pollinated silk sections were immunolabeled by LM20^22^, an antibody that preferentially recognizes methylesterified pectin. Compared with the apical region of *ga1* pollen tubes growing in the JKN^*Ga1-S*^ silks that exhibited significantly increased fluorescent signal intensity, those grown in the selfed JKN^*ga1*^ silks as well as the *Ga1-S* pollen tubes penetrating in JKN^*Ga1-S*^ and JKN^*ga1*^ silks exhibited weak intensity (Fig. 3d, e). This result suggested high DM pectin presenting in the *ga1* pollen tubes upon growing in *Ga1-S* silks, which may result from compromised PME activity. These findings also implied the impact of the female determinant on modifying the methylesterified pectin levels in the pollen tube walls. Hence, assembly of a PME complex that contains ZmGa1P and ZmPME10-1 is essential for modulating pectin properties to overcome the silk barrier of *Ga1*.

### Application of the *Ga1* locus in manipulation of pollen flow

**Fig. 4:**
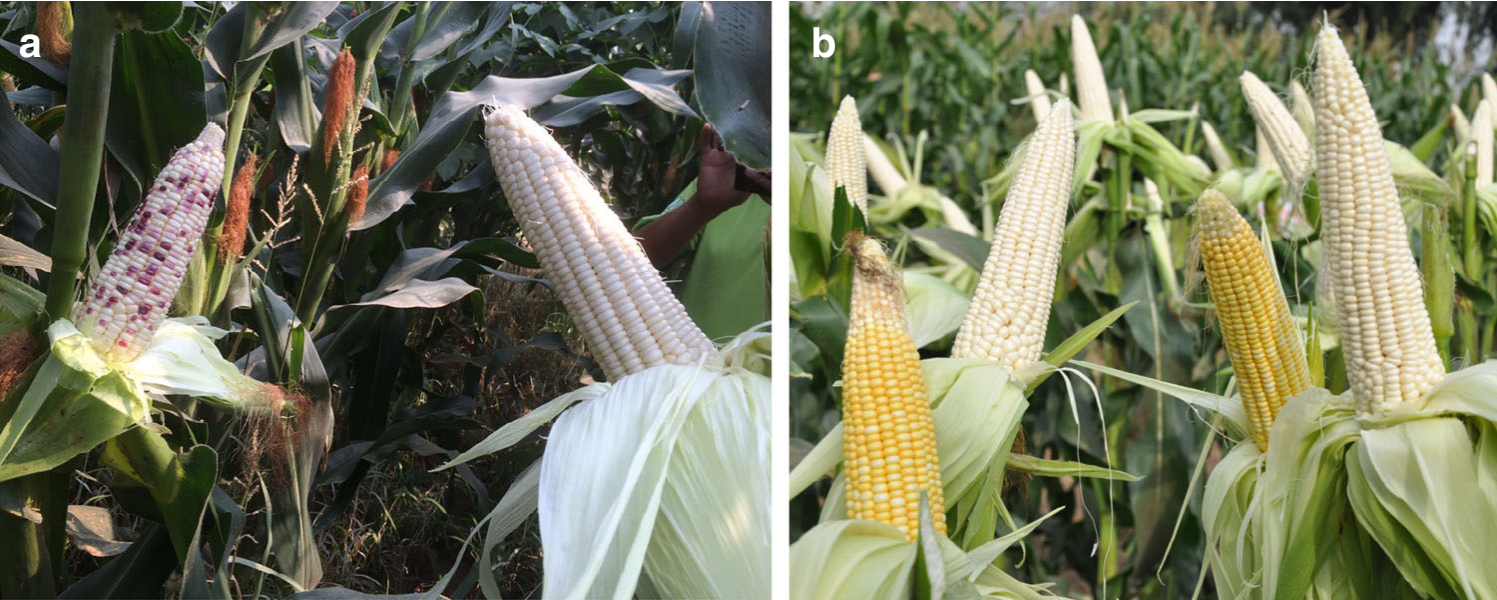
Field testing the isolation of *ga1* pollen from adjacent plants by *Ga1* locus. **a** KYN3^*ga1*^(left) and its near isogenic line KYN3^*Ga1-S*^ (right) grown surrounded by a *ga1* line with purple corn in Beijing, China. **b** KYN3^*Ga1-S*^ (white ears) was mingled with a *ga1* line with yellow corn in Jilin, China

UCI may provide a selective biological barrier to manipulate pollen flow among different categories of maize cultivars. Unlike popcorn and teosinte, the majority of dent and flint maize varieties carry the *ga1* haplotype^5^. Introduction of the *Ga1* locus into *ga1* lines may offer an effective method to isolate undesired pollen in commercial maize production. To validate the commercial value of the *Ga1* locus in dent and flint corn production, we developed a near isogenic white kernel hybrid KYN3 carrying *Ga1-S*. Based on field tests at two different locations for two years, this line rejected *ga1* pollen from adjacent plants without physical isolation as indicated by kernel colors (Fig. 4), demonstrating that the *Ga1* locus represents a feasible commercial carrier with significant market potential.

## Discussion

Incompatibility in flowering plants has a great impact on species evolution and resource exploitation. The maize *Ga1* locus has been studied for approximately one century, but low sequence homology between popcorn and conventional maize and difficulties in phenotyping and genotyping made cloning the determinants a daunting challenge^11^. In this study, despite more than 20,000 individuals were screened for recombinants, the mapping region was unable to be reduced less than 1.73 Mb, indicating genomic complexity at the *Ga1* locus even though it is not near the centromere. Combining with genome-wide association analysis, we cloned a *ZmGa1P* gene that performs the male function of the *Ga1* locus and demonstrated that ZmGa1P interacts with ZmPME10-1 to form a complex, which is essential for maintenance of the pectin methylesterification degree on the pollen tube walls for navigation through *Ga1-S* silks. Moreover, cloning of genes involved in UCI provides a tool for the control of pollen flow in commercial maize production and heterosis manipulation. Because *Ga1-M* can overcome *Ga1-S* barrier and this haplotype has been found at a much higher frequency in maize lines than previously thought^23^, its risk to *Ga1-S* hybrids should be aware.

Pectin deesterification catalyzed by PMEs can free carboxyl groups on pectin chains, promoting pectin crosslinking with Ca^2+^ and stiffening the cell wall^24^. PME activity can therefore modulate the pectin esterification-deesterification degree at the apical region of pollen tube walls and thereby affect the balance of plasticity and mechanical strength that is essential for pollen tube growth^17,20,21^. The role of PMEs in pollen tube growth regulation is well known; however, how PMEs control cross-incompatibility (CI) has not been reported. This study identified a pollen tube-specific PME, a type-II/group 1 PME (lacking a pro-region)^25,26^, as a pollen determinant of the *Ga1* locus in maize UCI. Another type-II PME, ZmPME3, has been reported to function as a female determinant candidate^15^. Maize potentially evolved a previously undescribed PME-mediated mechanism to control UCI, which is distinct from the known mechanisms that govern SI and CI in higher plants, such as the RNase-mediated system in Solanaceae, Rosaceae, and Plantaginaceae^12,13,27^ and the peptide ligand-kinase system in Brassicaceae and Papaveraceae^28–30^.

PME activity should be tightly controlled to fine-tune the pectin properties in specific regions of the walls, e.g., the pollen tube apex, to fit their roles in plant growth and development^20^. However, how male- and female-PME regulates pollen tube growth in UCI remains unknown. Differential expression patterns of PMEs and environmental conditions are key factors affecting PME activity. Here, we demonstrated that a pollen-specific PME is a male determinant, whereas the female determinant candidate appears to be a silk-specific PME^15^, providing crucial spatial and temporal information to unravel the intriguing action of both PMEs. However, other components involved in pectin configuration control cannot be excluded. In this study, biochemical analyses identified a PME complex, in which ZmPME10-1, a pro-region harboring type-I/group 2 PME^23,24^, represents one of the key components and interacts with ZmGa1P. Of note, the assembly of a PME complex found here is a previously undescribed mode for PME action. As an abundant pollen-specific PME, the function of ZmPME10-1 regulating pollen tube growth is expected because a mutation in VANGUARD1, a homolog isoform in Arabidopsis, exhibited abnormal pollen tube growth and male sterility^17^. Although overt PME activity has been detected in the pollen secretory proteins containing ZmPME10-1 or the ZmGa1P-ZmPME10-1 complex, an increase in methylesterified pectin that may result from compromised PME activity was only discovered in *ga1* pollen tubes upon growth in *Ga1-S* silks. We hypothesize that the pollen PME complex may interact with the female component to coordinate the final state of pectin methylation in pollen tube cell walls. However, further studies are required to clearly explore the intrinsic mechanism of *Ga1*-mediated UCI in maize.

Taken together, our findings open a door for understanding the molecular mechanisms underlying UCI in maize and likely other monocot plants^31,32^, which will contribute to crop molecular breeding.

## Methods

### Plant materials

The maize lines used in this study, including the association and mapping populations, were planted in the experimental fields of the Institute of Genetic and Developmental Biology (IGDB), Chinese Academy of Sciences (CAS) in Beijing and San Ya, Hainan province. The transgenic plants were grown in the fields and greenhouse with a 16-h light (25 °C)/8-h dark (18 °C) cycle. All plants were planted in plots at 0.6-m intervals in the horizontal direction and 0.4-m intervals in the longitudinal direction.

### Determination of the haplotype of *Ga1*

To determine the haplotype of *Ga1* in 1,299 inbred lines, SDGa25 (*Ga1-S*) was used as a female tester and planted in batches at 7-day intervals. Pollen from each of the 1,299 inbred lines was harvested and used to manually pollinate a set of three SDGa25 plants that were detasseled before flowering^19,33^. The resulting maize ear-florets (ears)^34^ with a full seed-set indicated bearing male function, whereas those with less than five seeds indicated a lack of male function. The identified inbred lines containing the male determinant gene were also used as females and were pollinated with B73 (*ga1*) pollen to test female function. The resulting ears with the full seed-set indicated a lack of female function, whereas those with less than five seeds indicated harboring female function. These tests were replicated at two different locations for two years. In total, 20 *Ga1-S* lines, 54 *Ga1-M* lines, and 872 *ga1* lines were determined (Supplementary Data 4). Genotyping *ZmGa1P* in the indicated *Ga1-S, Ga1-M*, and *ga1* lines was shown in Supplementary Figure 17.

### Fine-mapping of *ZmGa1P*

Two different BC_1_F_1_ segregating populations, *ga1* (B73 or J66)*//ga1* (B73 or J66)*/Ga1-S* (SDGa25) and *Ga1-S* (SDGa25)*//ga1* (B73 or J66)*/Ga1-S* (SDGa25), were used for mapping. The first population was developed using B73 or J66 as female and the F_1_ (B73 or J66 crossing SDGa25) plants as male. The resulting individuals in this BC_1_F_1_ population generated two types of gametes (*Ga1-S* or *ga1*). Additional phenotyping is required. To save labor, we produced the second population, in which SDGa25 (*Ga1-S*) lines were used as female and pollinated with pollen from the F_1_ (B73 or J66 crossing SDGa25) plants. Given the gametophytic nature of the male determinant, the resulting individuals in this BC_1_F_1_ population only had *Ga1-S* type gametes; thus, phenotyping is unnecessary^3^. In total, we used 21,600 individuals, including 5,700 from the first population and 13,943 from the second population, to screen the recombinants with the molecular markers (Supplementary Tables 4 and 5) designed according to the B73 sequence (RefGen_V4, www.maizegdb.org). Consequently, a total of 9 recombinants nearby the M53 marker on the distal side and 1 recombinant at the M16 marker on the proximal side were identified. The male determinant was ultimately mapped within a 1.73-Mb region.

### Genome-wide association analysis

More than 946 inbred lines containing *Ga1-S*, *Ga1-M*, and *ga1* haplotypes were employed to constitute a GWAS panel. DNA was extracted from five seedlings of each line and genotyped using the Axiom Maize Genotyping Array consisting of sequence information from 616,201 variants. The high-density SNP chip has been successfully applied for association mapping and comparative genomics^35^. Quality control was performed by limiting the MAF (minor allele frequency) value per SNP to less than 0.05, the missing rate per SNP to less than 0.1 and the missing rate per individual to less than 0.1. After filtering, 465,112 polymorphic SNPs and 875 individuals (19 *Ga1-S*, 42 *Ga1-M*, and 814 *ga1* lines) qualified for association analysis. The phenotype values of each inbred line in the association test were assigned 1 or 0 to represent cross-compatible and incompatible, respectively. To examine the association between SNPs and the traits, Tassel 3.0 with a compressed mixed linear model (CMLM) was employed for GWAS^36–38^ as this model considers the population structure (Q) and familial relationship (K). The analysis was conducted with the GAPIT package in R^39^. The population structure was analyzed by principle component analysis (PCA). The first four principal components of PCA were used as a covariate matrix for the CMLM as fixed effects. The relative K was incorporated as a random effect in the CMLM, calculated by the VanRaden method^40^. The threshold for significant association (*P* < 2.15 × 10^−8^) was corrected using the Bonferroni correction method (*P* = 0.01/*n*, 0.01 was the type I error rate, and n was the total number of markers used for the association test)^41^. To identify the candidate genes underlying the association signals, we performed linkage disequilibrium (LD) analysis on each chromosome and plotted the whole genome LD decay against physical distance. The average distance over which LD decayed to half of its maximum value in our association panel was approximately 100 kb. Among the identified unique association signals, the annotated genes within 200 kb of the lead SNP in the mapping interval were validated and regarded as the candidate gene. The corresponding positions of each SNP were mapped based on the reference sequence *B73 AGP_v4*^16^.

### Construction and screening of the SDGa25 BAC library

To construct a BAC library of SDGa25, high-molecular weight genomic DNA was extracted from leaf tissues, partially digested by the restriction enzyme *Hin*dIII, and ligated into the vector pIndigoBAC536-S^42,43^. The ligation products were transformed into ElectroMAX DH10B T_1_ phage-resistant cells (Invitrogene) and screened on LB medium with chloramphenicol (12.5 mg/L), 5-bromo-4-chloro-3-indolyl-β-d-galactopyranoside (80 mg/L) and isopropyl β-d-1-thiogalactoside (100 mg/L). White colonies were individually picked into 384-well microtiter plates. In total, 207,260 BAC clones were arranged in 540 384-well plates, representing a 10-fold coverage of the maize genome. We developed a PCR-based method to screen clones harboring the pollen determinant. A 384-well stock was duplicated and pooled to extract a primary DNA pool using the ZR BAC DNA Miniprep Kit (Zymo Research Corporation, Irvine, CA, USA). In total, 540 primary DNA pools were prepared and screened using the primers listed in Supplementary Table 6.

BAC DNA sequencing was performed using PacBio SMRT sequencing technology^44^ by the Nextomics Biosciences Company (Wuhan, China). In short, SMRTbell DNA template libraries with an average ~17-kb insert size for the BAC sample were prepared from sheared DNA by G-tubes (Covaris) according to the manufacturer’s recommendations. The library was bound with P6 polymerase, and complexes were then loaded onto SMRT cells (version V3). SMRT sequencing was performed on PacBio RSII using C4 sequencing reagents according to standard protocols. Movie lengths were 240 min for the SMRT cell. In total, one SMRT cell yielded output data of 1,376,923,513 bp with an average coverage of ∼4653× and an average length of 7688 bp subreads. To assemble the BAC sequence, clean reads obtained by removing adapters and low-quality reads (reads length <500 bp, quality <0.8) were assembled de novo using the RS hierarchical genome assembly process (HGAP) protocol version 2.3.0^45^. Finally, a 154,617-bp gapless completed circular sequence was assembled and trimmed into clean data of 135,485 bp with 44.5% GC content. Annotation was performed using the FGENESH gene annotation program (www.softberry.com)^46^. Interspersed repeats and low-complexity DNA sequences were screened using the RepeatMasker program (http://www.repeatmasker.org/).

### Generation of maize transformation plants

For the genomic complementary assay, the 6,287-bp *Zm00001d048936* genomic fragment containing the coding region and the 2,980-bp promoter and 2,046-bp terminator regions from SDGa25 was inserted into pCAMBIA3300 to generate the genomic construct. In addition, the *ZmGa1P* coding sequence was amplified from SDGa25 and inserted into the pCAMBIA2300 vector between the maize ubiquitin promoter and the nopaline synthase terminator to generate an OE construct. The resulting constructs were introduced into the wild-type inbred line B104 (the genomic construct) and Z31 (the OE construct) through *Agrobacterium tumefaciens* infection, separately. Positive transgenic events were identified using the primers presented in Supplementary Table 6. Pollen from *T*_0_ to *T*_2_ transgenic plants was collected to fertilize SDGa25 plants to test cross-compatibility. At least five individuals from each transgenic event and the corresponding nontransgenic sibling plants were selected to provide pollen, and maize ears from at least three SDGa25 plants were used as the female testers. To examine the function of *ZmGa1P* in the female barrier, the transgenic plants were used as the female to pollinate B73 pollen. These crosses were evaluated by examining the seed-set on the resulting ears. Genotyping results on the seeds of parents and the indicated cross-pollinations were shown in Supplementary Figure 17, 18.

### Gene expression analyses

To examine the expression pattern of the candidate genes located within the mapping region, roots, stems, leaves, anthers, and silks were harvested from B73 and SDGa25 plants at the proper growth stages. Total RNA was isolated from the different maize tissues using TRIzol (Invitrogen) and treated with RNase-free DNase I (Thermo Fisher Scientific) to remove DNA according to the manufacturer. Complementary DNA (cDNA) was synthesized from 2 μg of total RNA using RevertAid reverse transcriptase (Thermo Fisher Scientific) with the oligoT_18_ primer. For PCR analysis, cDNAs were used as template and amplified with the primers shown in Supplementary Table 6 (26 cycles of 95 °C for 30 s, 60 °C for 30 s, and 72 °C for 15 s). RT-PCR examinations of the candidate gene expression in pollen were shown in Supplementary Figure 16. The expression profile of *ZmPME10-1* was also analyzed based on online public RNAseq data^47^. Three biological replicates were tested.

To determine the expression level of *ZmGa1P* in various inbred lines or transgenic and nontransgenic plants, mature anthers were collected from 20 of the *Ga1-S*, *Ga1-M*, and *ga1* lines and the transgenic plants. Total RNA was extracted from anthers as described above. After synthesizing cDNA, quantitative PCR (qPCR) was performed on a LightCycler 480 Real-Time PCR System (Roche Diagnostics) with SYBR Green Master mix (45 cycles of 95 °C for 10 s, 60 °C for 30 s, and 72 °C for 10 s). *ZmGAPDH* was used as an internal control and the 2^−ΔΔCt^ method was used to calculate the relative gene expression level. At least three biological replicates were performed.

### Pollen isolation

To harvest the pollen used in this study, tassels were vigorously shook and covered with paper bags in the evening before pollen harvest^33^. Pollen grains were collected by shaking the tassels in paper bags at 9:30 a.m. and 2:00 p.m. The fresh pollen grains were used for pollination and in vitro germination.

### In vivo pollen-tube growth assay

B73 and SDGa25 used for in vivo pollen-tube growth analysis were grown in experimental fields for self-crossing and reciprocal crossing^11^. Six hours after pollination, silks were collected and fixed in 4% paraformaldehyde in 50 mM sodium phosphate, pH 7.0 (PBS) for at least one day. The fixed silks were washed with the above buffer and immersed in 1 M NaOH overnight for softening. The pistil was then stained with 0.1% aniline blue (Amresco) in 0.1 M potassium phosphate (pH 8.0) for 6 h. The samples were observed under an Axio Skop2 microscope (Zeiss) equipped with an ultraviolet filter. The length of pollen tubes was evaluated between the pollen entry place and the tip of pollen tubes using ImageJ software (http://rsb.info.nih.gov/ij/). Twenty-four pollen tubes from at least three plants were examined in each cross.

### Detection of ZmGa1P in the in vitro germinated pollen tubes

Fresh pollen harvested from the *Ga1-S* plants and wild-type lines was sprayed onto the germination medium (10% [w/v] sucrose, 0.0005% [w/v] H_3_BO_3_, 10 mM CaCl_2_, 0.05 mM KH_2_PO_4_, and 6% [w/v] polyethylene glycol 4000) in petri dishes immediately^48^. After germination at room temperature for 20–60 min, pollen tubes were collected by precipitation and fixed with 4% paraformaldehyde in PBS. For immunolabeling of ZmGa1P at pollen tubes, a rabbit antibody was generated using a polypeptide (70–83 aa) of ZmGa1P as an immunogen. The fixed pollen tubes were extensively rinsed in PBS and then probed with the purified primary antibody of ZmGa1P at a 1:1000 dilution. Fluorescein isothiocyanate (FITC) coupled with anti-rabbit IgG (Sigma, F0382) was used as a secondary antibody at a 1:500 dilution. Fluorescence signals were recorded using a confocal laser scanning microscope (Axio imager Z2, Zeiss). Western blot of secretory proteins from pollen of the maize lines was shown in Supplementary Figure 19.

### Scretome assay

Supernatant of the medium after germination of SDGa25 and J66 pollen was collected by centrifugation at 200*×g* at room temperature to remove pollen. The supernatant samples that contained secretory proteins were loaded onto a HiTrap Q HP column (GE) equilibrated with buffer A (20 mM Tris-HCl, pH 7.5) and fractionated with a linear gradient of 0–1 M NaCl in buffer A after extensive washing in the FPLC system (ÄKTA pure, GE). Proper amounts of each eluent fraction were subjected to sodium dodecyl sulfate polyacrylamide gel electrophoresis (SDS-PAGE) for staining with Coomassie Brilliant Blue G-250 (CBB) or for western blotting by probing with anti-ZmGa1P antibody (at 1:500 dilution) to monitor the presence of ZmGa1P proteins. The CBB stained gels of fraction 6–10 were further subjected to liquid chromatography tandem-mass spectrometry (LC-MS/MS) analyses^49^. In brief, the gel slices were subjected to an in-gel digestion with trypsin. After extraction with 60% acetonitrile, the resultant peptides were separated on a reverse phase C18 column and detected with a linear ion trap mass spectrometer (Thermo Finnigan). The generated mass spectra data were analyzed with Proteome Discoverer (Thermo Fischer Scientific) and were summarized in the Supplementary Table 3 and Supplementary Data 2 if the peptide spectrum match (PSM) was greater than 3. Three biological replicates were tested. Western blots and CBB stained SDS-PAGE gels of secretory proteins from pollen of maize lines were shown in Supplementary Figure 20.

### Pectoplate assay

Pectin methyl esterase activity was analyzed based on the report^50^. In brief, the agarose plate was prepared by pouring 50 mL of media containing 0.1% (w/v) citrus pectin (Sigma), 1% (w/v) LE agarose (USB), 12.5 mM citric acid, and 50 mM Na_2_HPO_4_, pH 6.5, into 12-cm^2^ petri dishes. After solidification, the plates were punched using a capillary tube at equal distance. Equal volumes of the eluent fractions were loaded into the punched wells and incubated at 30 °C for 10–16 h. The plates were then stained with 0.05% (w/v) ruthenium red (R2751, Sigma) for 30 min and destained by rinsing with distilled water. The stained circle size indicated PME activity. Three biological replicates were tested. Pectoplate assays were shown in Supplementary Figure 20.

### Quantification of PME activity

To quantify PME activity, different amounts of fractions were added to the reaction solution (0.01 U/μL alcohol oxidase, 0.5% citrus pectin, 200 mM sodium phosphate, pH 6.2) and incubated at room temperature for 30 min. Fluoral-P (Sigma) was then added to a final concentration of 4 mg/mL. After incubation at room temperature for 5 min, the fluorescent intensity was measured in an Enspire reader (PerkinElmer) at 510 nm upon excitation at 405 nm. The released methanol amounts that represent PME activity were quantified based on the standard curve established using a methanol gradient. Three biological replicates were tested.

### DSS crosslink assay

To determine the relationship between ZmGa1P and ZmPME10-1, fraction 8 of pollen secretory proteins of SDGa25 and J66 pollen was desalted with a PD MiniTrap G-10 column (GE) in 50 mM hydroxyethyl piperazineethanesulfonic acid buffer (pH 8.0) and concentrated in a filtrate column (Amicon, Millipore). The conditioned samples were cross-linked by adding disuccinimidyl suberate (DSS, Sigma) at a 75-fold molar excess. After incubation for 1 h at room temperature, the reaction was quenched by adding 1 M Tris (pH 8.0) to a final concentration of 50 mM. The samples were separated on a NuPAGE 4–12% Bis-Tris gel (Invitrogen) and subjected to western blotting using the antibody against ZmGa1P at 1:500 dilution. For mass spectrometry analyses, the ~100-kD bands separated by SDS-PAGE were stained with CBB and excised for LC-MS/MS analyses as described above. This experiment was also performed using the total proteins extracted from unpollinated and pollinated SDGa25 silks. Three biological replicates were tested. Western blots for crosslinking proteins were shown in Supplementary Figure 21.

### Firefly luciferase complementation assay

To examine protein-protein interactions, a split luciferase complementation assay was employed^51^. The coding sequence of *ZmGa1P* without a signal peptide (82−1,137 nt) was amplified from SDGa25 and fused to the pCAMBIA-35S-CLuc vector. Simultaneously, the coding region without a signal peptide (88−2,184 nt) and the PME domain (1210−2187 nt) of ZmPME10-1 were amplified from pollen of SDGa25, and inserted into vector pCAMBIA-35S-NLuc, respectively. The resulting constructs were transformed into *A. tumefaciens* and cultivated overnight. The *Agrobacterium* samples were resuspended in the infiltration medium containing 10 mM MgCl_2_, 10 mM 2-(N-morpholino) ethanesulfonic acid (MES), and 200 mM acetosyringone, and incubated for 1–3 h at room temperature. For co-infiltration, equal volumes of *Agrobacterium* containing the NLuc- or CLuc-fused construct were pairwise mixed at OD_600_ of 1.6 to 1.8 and infiltrated the abaxial side of 4- to 6-week-old *N. benthamiana* leaves. After infiltration, plants were cultivated in the dark for 12 h and then moved into a greenhouse with a 16-h light/8-h dark cycle. The infiltrated leaves were cut approximately 48 h later and sprayed with 1 mM luciferin. Images were captured using a low-light cooled CCD imaging apparatus (CHEMIPROHT 1300B/LND, 16 bits; Roper Scientific). The signal peptide cleavage sites of the above proteins were predicted using the SignalP 4.1 server (http://www.cbs.dtu.dk/services/SignalP/)^52^. Three biological replicates were tested.

### Immunolabeling analysis

To visualize the difference of pectin esterification in pollen tubes grown in silks, two near isogenic lines that vary in *Ga1* locus, JKN^*Ga1-S*^ and JKN^*ga1*^, were developed by a series of backcrosses. The silks of JKN^*Ga1-S*^ and JKN^*ga1*^ that pollinated with themselves or with reciprocal pollen were fixed in 4% paraformaldehyde. After dehydration with an ethanol gradient, the silks were paraffin embedded. Ten-micrometer thick longitudinal sections were prepared using a microtome (RM2235, Leica). For immunolabeling, the silk sections were dewaxed, rinsed in PBS and probed with LM20^22^, an antibody against highly esterified homogalacturonan (University of Leeds, UK), at a 1:20 dilution. FITC-coupled anti-rat IgG (Sigma, F1763) was used as a secondary antibody at a 1:500 dilution. Fluorescence signals were recorded with a confocal laser scanning microscope (Axio imager Z2, Zeiss). In this experiment, silks from at least three individual plants were employed for analysis.

### Field tests of the *Ga1* locus in pollen isolation

A marker-assisted backcrossing (MAB) strategy was employed to introgress the *Ga1* locus from SDGa25 into the elite parental lines of the KYN3 hybrid with white kernels. KYN3 parental lines were crossed with SDGa25 to produce the first generation, which was further backcrossed with KYN3 parental lines eight times using a single-seed descent approach. Three polymorphic markers M14, M1, and M10 (Supplementary Table 4) located in the *ZmGa1P* interval were used to select the *Ga1* locus during backcrosses. After nine rounds of backcrossing, individuals carrying the *Ga1* locus were selfed. The near isogenic hybrids carrying homozygous *Ga1* locus JKN3^*Ga1-S*^ and wild-type JKN3^*ga1*^ were subjected to field tests in the farms located in Beijing and Jilin, China, respectively. The near isogenic hybrids grown in the fields were surrounded by or mingled with *ga1* hybrids that produce yellow or purple kernels without physical isolation. The outcrossing with the pollen from adjacent plants was evaluated by the kernel color on the resulting ears.

## Electronic supplementary material

Supplementary Information

Description of Additional Supplementary Files

Supplementary Data 1

Supplementary Data 2

Supplementary Data 3

Supplementary Data 4

## Data Availability

The authors declare that all other data supporting the findings of this study, as well as vectors and plant seeds generated as part of this study, are available from the corresponding authors upon request.
