# Supplementary material for: A PECTIN METHYLESTERASE gene at the maize Ga1 locus confers male function in unilateral cross-incompatibility
Source: Nat Commun. 2018 Sep 10;9:3678. doi: 10.1038/s41467-018-06139-8 (PMC6131150; doi:10.1038/s41467-018-06139-8)
Supplement: Supplementary file 1 — Supplementary Information [file 41467_2018_6139_MOESM1_ESM.pdf]

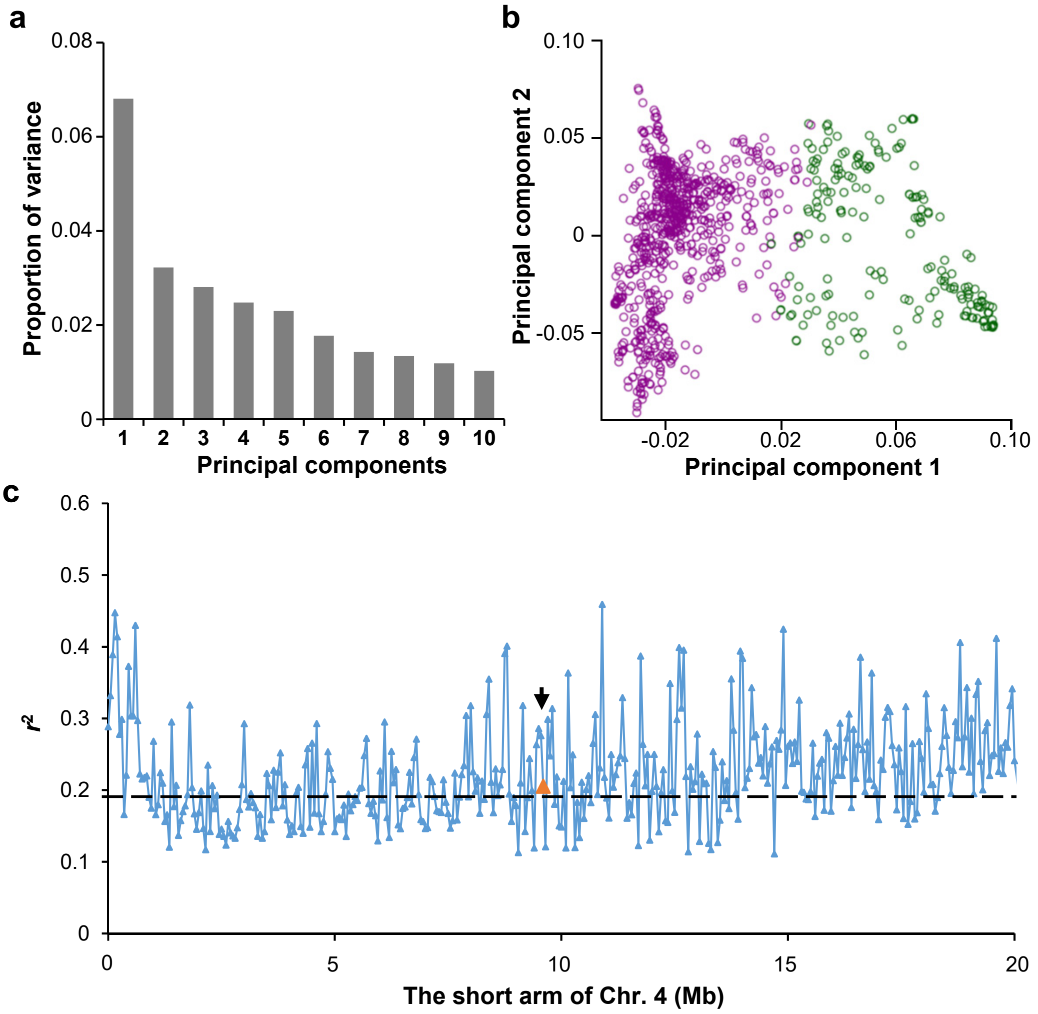

Supplementary Figure 1 Population structure analysis. (**a**) Top-10 principal components explained variations observed in the association panel. (**b**) Principal component plot, to show that principal component 1 explained the genomic variation between the inbred lines from temperate (purple) and tropic (green) regions. (**c**) Linkage disequilibrium plot of the first 20 Mb in Chromosome 4 with sliding windows of 50 kb, showing that the pollen determinant (indicated by a brown dot and a black arrow) is located in a LD region less than 200 Kb.

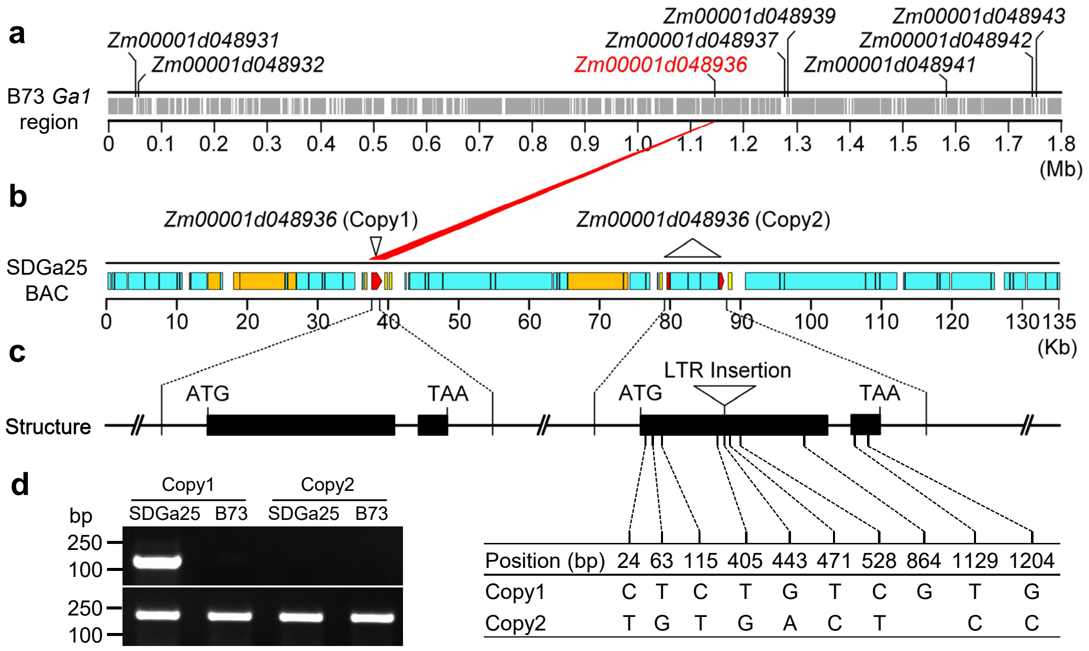

Supplementary Figure 2 Analysis of the BAC clone harboring *Zm00001d048936* of *Ga1-S*. (**a**) Mapping region of the pollen determinant. The gray boxes indicate the locations of transposons and retrotransposons. The candidate gene is indicated by red letters. (**b**) Annotation of the BAC sequence. Red arrows represent two copies of *Zm00001d048936.* LTR/Gypsy is indicated by light-blue boxes. LTR/Copia is indicated by light-yellow boxes. DNA/CMC-EnSpm is indicated by gray boxes. (**c**) Gene structure and polymorphic sites of two copies of *Zm00001d048396*. Exons and introns are indicated as black rectangle bars and lines, respectively. Polymorphic sites are positioned relative to the start codon. (**d**) RT-PCR to examine the expression of two copies of *Zm00001d048936* in SDGa25 (*Ga1-S*) and B73 (*ga1*) pollen. *ZmGAPDH* was used an internal control (bottom panel).

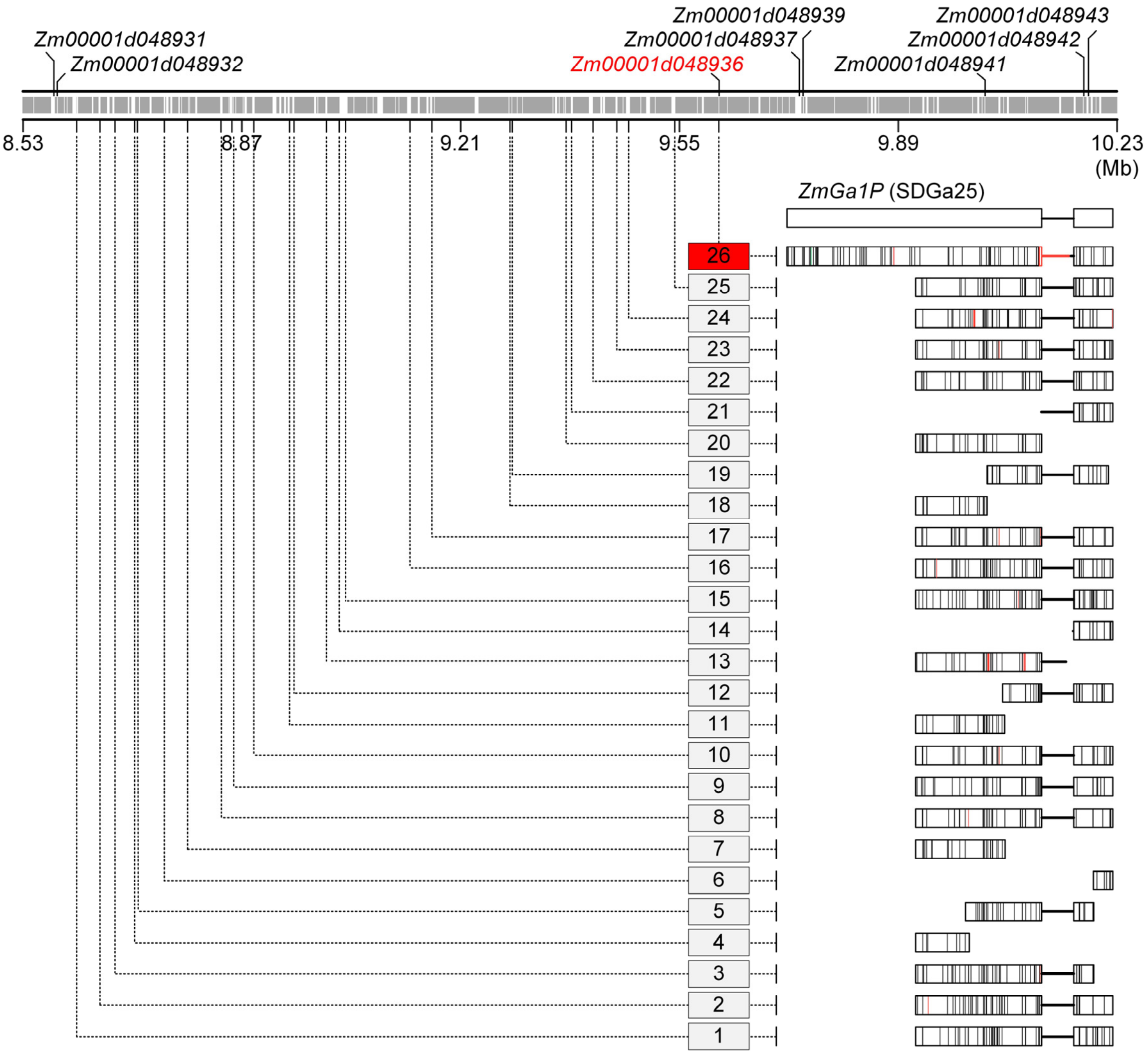

Supplementary Figure 3 Gene structure of *ZmGa1P* copies in the mapping region of B73. The red box indicates full-length *Zm00001d048936/ZmGa1P* in B73. The gray boxes indicate partial PME fragments. The black and red lines in the gene structures represent the SNPs and InDels compared with SDGa25.

```
SDGa25 ATGATGATGAGTAAACAAATGCTCGTCTTGTCCCTGCTCCTAGTGTTGTTCGAGCTTGGATCTCTGCCGACGACATCGTGCAAAAAG--- 87
B73    ATGGTGATGAGTAAACACATGCTCATATTGTCCCTGCTCCTAGTGTTGTTCGAGCTTGTATCGCTACCGACGACATCGTGCAATAGGGTC 90
       *** ************* ****** * ******************************* *** ** ***************** * *

SDGa25 GTCTTTTTCAACTTATGGGTGACAAACCAGCCAGCTAATGCCACCCAAGATGCGGGGTGTGCTAAGAAAGATGATGCGCTCTCCTCCGCC 177
B73    GTCTCTTTCAACTCATGGGTGAGACACCAACCAGCTAATGCCACCCAAGATGCGGGGTGTGCTAAGAAAGATGATGCGCTCTCCTCTGCC 180
       **** ******** ******** * **** ******************************************************** ***

SDGa25 GACACCATTAAGGTATGGAATTACATCGACCCTGCCTCTCAATTGAGACCTGAAGATGGCGGTTACACGACCATTAGCGAGTCCATCGCC 267
B73    AACACCATTAAGGTAAGGAATTACATTGACCCTGCCTCCGAATTGAGACCTGAAGATGGTGGTTACACGACCATTAGCGAGTCCATTGCC 270
        ************** ********** ***********  ******************* ************************** ***

SDGa25 AACATCCCTGAGGACAACGCCAAACGCTACCTCCTTATCCTCAAACCTGGTGTTGTGTTCCGCGAGAAGCTGTTACTCGGTAGAAGCAAG 357
B73    AACATCCCTGACGACAACACCAAATGCTACGTCCTTACCCTCAAACCTGGTGTTGTGTTCCGCGAGAAGCTGTTACTCGGTAGAAGCAAG 360
       *********** ****** ***** ***** ****** ****************************************************

SDGa25 CCTTTCATCACCATAATGTCCGAGGACCCCATGAACCCTGCTGTTATCGTCTGGAATGACACTGCCACCACCATGGGCAAGGACGGCAAG 447
B73    CCATTCCTCACCATAATATCCGAGGACCCCATGAACCCAGCTATTATCGTCTAGAATGACACTGCCACCACCATGGGCAAGGACGGCAAG 450
       ** *** ********** ******************** *** ********* *************************************

SDGa25 CCCCTTGGTGTGGATGGAAGCAGTACCATGGCCATAGAGTCCGACTATTTTGTCGCCTACAACGTTGTCTTCAAGAACGACGCGCCGCTA 537
B73    CCCCTTGGTGTGGATGGAAGCAGCACCATGGCGATAGAGTCCAACTATTTTGTCGCCTACAACATTGTCTTCAAGAACGACGCGCCGCTA 540
       *********************** ******** ********* ******************** **************************

SDGa25 CCAAAGCTAGGGGAAAAGAAAGGTGAGGCACCAGCACTGCGAGTGATGGGAACAAAGGCAACCTTCTACAATTGCACCATCGAAGGCGGC 627
B73    CCAAAGCTAGGGGAAAAGAAAGGTGAGGCACCAGCACTGCGAGTGATGGGAACAAAGGCAACCTTCTACAATTGCACCATCCAATGCGGC 630
       ********************************************************************************* ** *****

SDGa25 CAGGGTGCTCTGTACGACCAGACGGGTCTGCACTACTTCAAGGCTTGTGCCATCAAGGGAACCATCGACTTCATCTTCGGATCTGCCAAG 717
B73    CAGGGTGCTCTAAACGACCAGACGGGTCTGCACTACTTCAAGGCATGTGCCATCAAGGGAACCATCGACTTCATCTTCGGATCTGCCAAG 720
       ***********  ******************************* *********************************************

SDGa25 TCATTTTATGAGGAATGCAAAATCGTTTCGGTGTTGAAGGAGGCATTGGTATTGCCATTGGCACCACCGGAGCAGGACCGCTCTAGAAAT 807
B73    TCATTTTATGAGGAATGCAAAATTGTTTTGGTGTTGAAGGAGGCATTGACATTGCCAATCGTGCCACCGGAGCAGGACCGGTCTAGAAAT 810
       *********************** **** *******************  ******* * *  ***************** *********

SDGa25 CCCATCGAAATCGCCCCAGGCAAGAGCGGGTTGGCATTCAAGACTTGCACAATCGAGGGGGAAGGAGAAAAAATTTACTTGGGTAGGGTG 897
B73    CCCATCAAAATTGCCCCAGGCAAGAGCGGGTTGGCATTCAAGACTTGCACAATCGAGGGGGAAGAAGAAAAAATTTACTTGAGTAGGGTG 900
       ****** **** **************************************************** **************** ********

SDGa25 GGCACGCCTGTGATATACTCCTACACTAATATAGGTAAGGAGATTGTAGGCATAATATCTGATGGTCGGGACGTCCAGACAGTCGAAAGG 987
B73    GGCACGCCTGTGATCTACTCCTACACCGATATAGGTAAGGAGATTGTAGGCATAATATCTAATGGTTACGACGT---------------- 974
       ************** ***********  ******************************** *****   *****

SDGa25 TACCACTCCTATGTCCATTACATCCTCTCCTTTCTTCATATATGATTGTGTGATTAAAGGTGTTGTTCATTATCTATACTGATGAAAGGT 1077
B73    ------------------------------------------------------------------------TGTATACTAAAGAAAGG- 991
                                                                               * ****** * ******

SDGa25 GTTGTTGTTGCGTTGCATT-TTTTATTATAGGGGGTACTACTGCGCCACTTTTAGGTGTTACGGGCCTGGGATGTCTCCAATGGTAACCT 1166
B73    --TGTTGTTGCGTTGCATTCTTTTTTTATAGGGGGTACTATTGCGCCACTTTCAAGTGTTACGGGCCTAGGATGTCTCCAATGGTAACCT 1079
         ***************** **** *************** *********** * ************* *********************

SDGa25 CAACTCTGACCTATGTCGAGGCAATACCCTTTCTCGGGATACACTACATCTCGGGGGAGTCATGGATCCCGTCCCTACCACCCGCTGAAG 1256
B73    CAACTCTAACCTATGTCCAGGCAATACCCTTTCTCGGGATACACTACATCTCGAGGGAGTCGTGGATCCCGTCCCTACCACCCGCTGAAG 1169
       ******* ********* *********************************** ******* ****************************

SDGa25 AATAA 1261
B73    AATAA 1174
       *****
```

Supplementary Figure 4 Alignment of the *ZmGa1P* genomic sequence between SDGa25 and B73. The two exons in SDGa25 (*Ga1-S*) are highlighted in gray. * indicates the identical nucleotides.

```
SDGa25 MMMSKQMLVLSLLLVLFELGSLPTTSCKK-VFFNLWVTNQPANATQDAGCAKKDDALSSADTIKVWNYIDPASQLRPEDGGYTTISESIA 89
B73    MVMSKHMLILSLLLVLFELVSLPTTSCNRVVSFNSWVRHQPANATQDAGCAKKDDALSSANTIKVRNYIDPASELRPEDGGYTTISESIA 90
       *.*** ** ********** *******   * ** **  ********************* **** ******* ****************

SDGa25 NIPEDNAKRYLLILKPGVVFREKLLLGRSKPFITIMSEDPMNPAVIVWNDTATTMGKDGKPLGVDGSSTMAIESDYFVAYNVVFKNDAPL 179
B73    NIPDDNTKCYVLTLKPGVVFREKLLLGRSKPFLTIISEDPMNPAIIV------------------------------------------- 137
       *** ** * * * ******************* ** ******** **

SDGa25 PKLGEKKGEAPALRVMGTKATFYNCTIEGGQGALYDQTGLHYFKACAIKGTIDFIFGSAKSFYEECKIVSVLKEALVLPLAPPEQDRSRN 269
B73    ------------------------------------------------------------------------------------------ 137

SDGa25 PIEIAPGKSGLAFKTCTIEGEGEKIYLGRVGTPVIYSYTNIGKEIVGIISDGRDVQTVERGYYCATFRCYGPGMSPMVTSTLTYVEAIPF 359
B73    ------------------------------------------------------------------------------------------ 137

SDGa25 LGIHYISGESWIPSLPPAEE 379
B73    -------------------- 137
```

Supplementary Figure 5 Alignment of ZmGa1P protein sequence between SDGa25 and B73. The underlined amino acid sequence is used to generate the antibody. * indicates the identical amino acids.

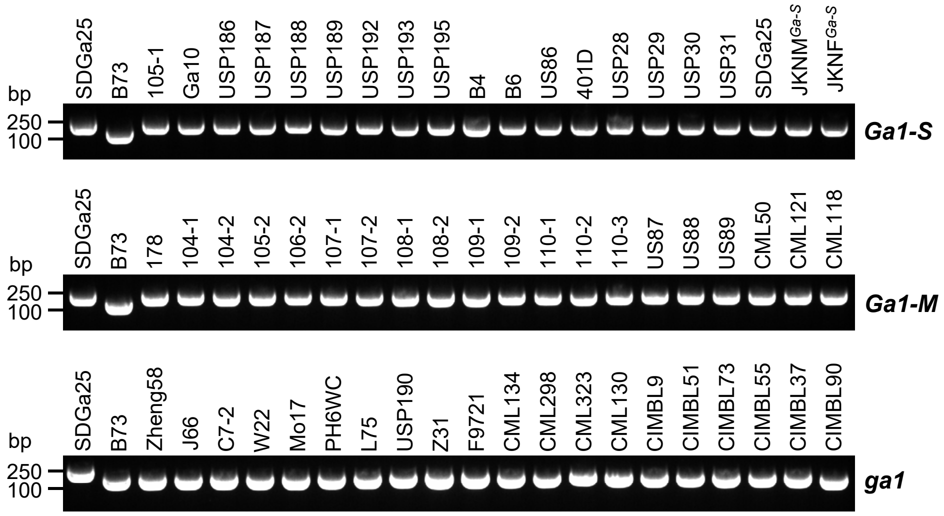

Supplementary Figure 6 Genotyping *ZmGa1P* in *Ga1-S, Ga1-M*, and *ga1* lines. A total of 946 inbred lines were genotyped, including 20 *Ga1-S* lines, 54 *Ga1-M* lines and 872 *ga1* lines, using a marker (ZmGa1P-Del) that showed the 88-bp deletion in *ga1* plants. Genotypes of 20 representative lines of *Ga1-S* (upper panel), *Ga1-M* (middle panel), and *ga1* (lower panel) are shown.

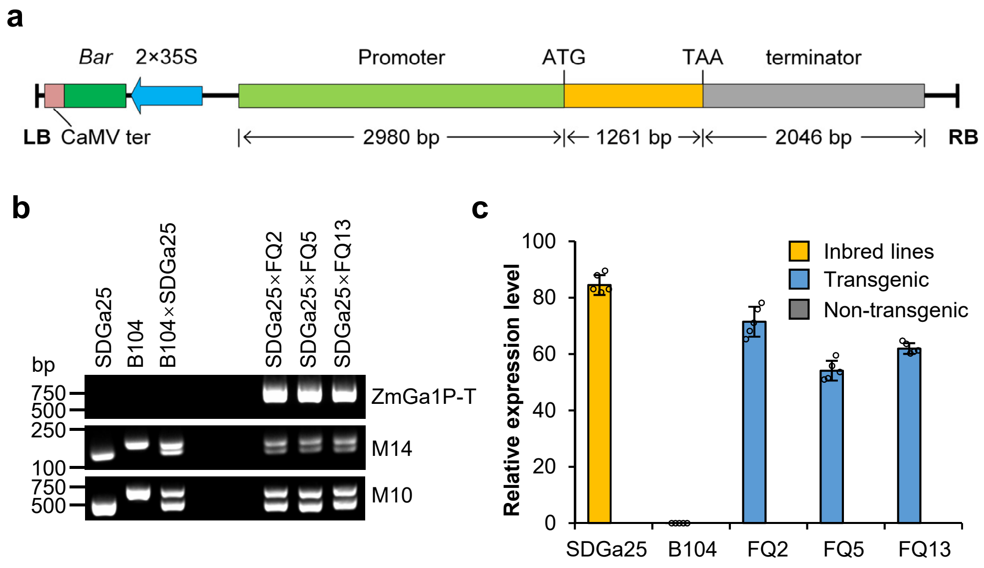

Supplementary Figure 7 Validation of *ZmGa1P* by expressing its genomic fragment. (**a**) The construct containing *Zm00001d048936/ZmGa1P* genomic fragment cloned from SDGa25 (*Ga1-S*). LB, left border; RB, right border; CaMV ter, cauliflower mosaic virus polyadenylation signal; *Bar,* Basta-resistance gene. (**b**) Genotyping the seeds of parents and indicated cross-pollination. Marker ZmGa1P-T was used to confirm the transgenes; M14 and M10 markers were used to check heterozygosity. (**c**) Quantitative analysis of *Ga1-S* type *Zm0001d048936/ZmGa1P* transcripts in pollen of the indicated plants. FQ2, FQ5, and FQ13 represent transgenic plants that expressed *Zm0001d048936/ZmGa1P* (genomic gene) in B104 (*ga1*). Error bars represent the means ± SD (n = 5).

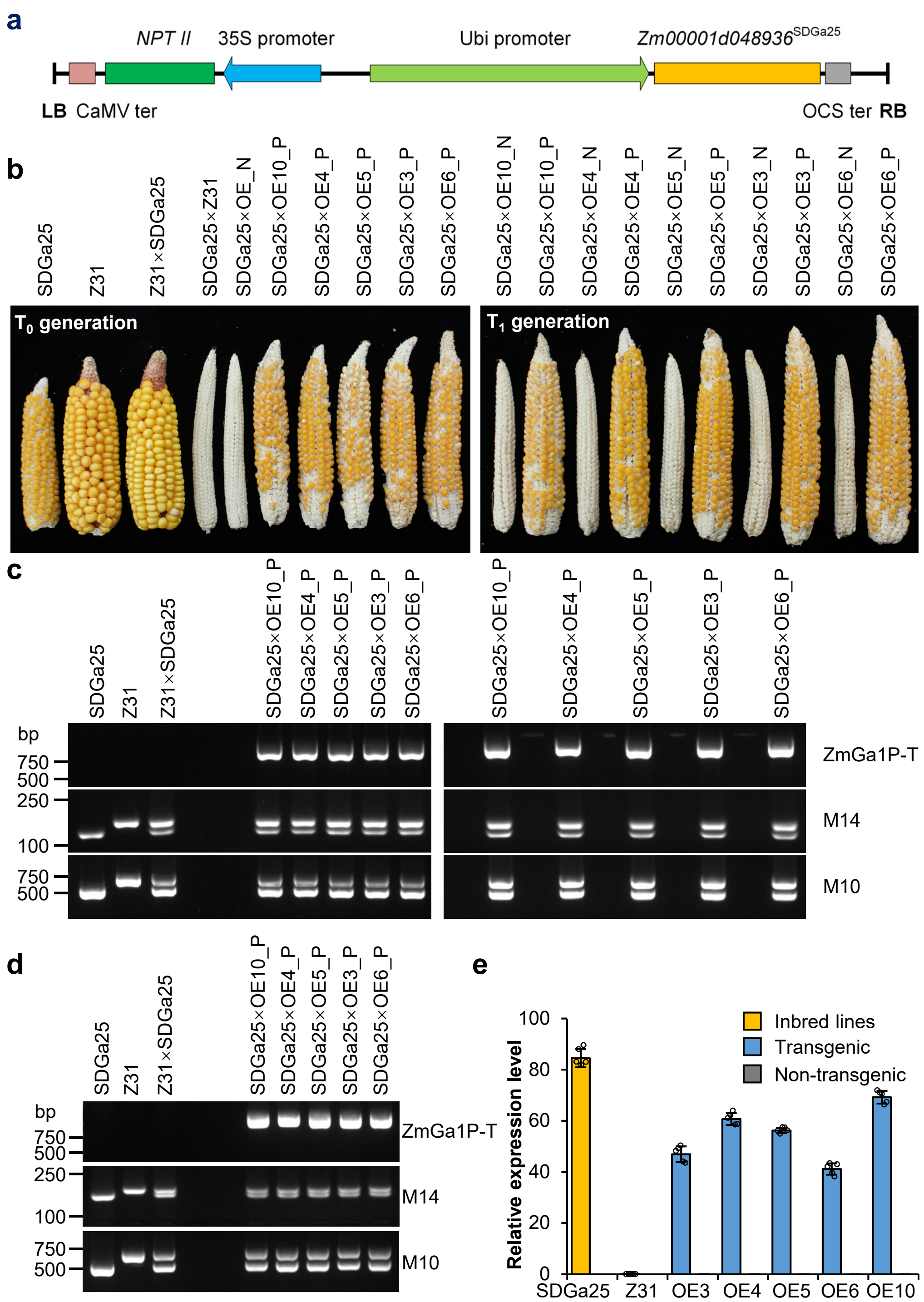

Supplementary Figure 8 Validation of *ZmGa1P* by overexpression. (**a**) The construct for overexpressing *Ga1-S* type *Zm00001d048936/ZmGa1P*. LB, left border; RB, right border; Ubi, ubiquitin; 35S, cauliflower mosaic virus 35S promoter; *OCS* ter, octopine synthase gene terminator; CaMV ter, cauliflower mosaic virus polyadenylation signal; *NPT* II, neomycin phosphotransferase II resistance gene. (**b**) Cross-compatibility analysis, to show the ears of SDGa25 (*Ga1-S*) pollinated by Z31 (*ga1*) and the transgenic plants (OE_P) at T_0 (left) and at T_1 generation (right). The corresponding nontransgenic plants (OE_N) were used as negative controls. (**c** and **d**) Genotyping the seeds of parents and indicated cross-pollinations by using pollen from T_0 and T_1 plants (**c**) and from T_2 plants (**d**). Marker ZmGa1P-T was used to confirm the transgenes; M14 and M10 markers were used to check heterozygosity. (**e**) Quantitative analysis of *Zm0001d048936/ZmGa1P* transcripts in pollen of the OE transgenic plants in Z31 background. Error bars represent the means ± SD (n = 5).

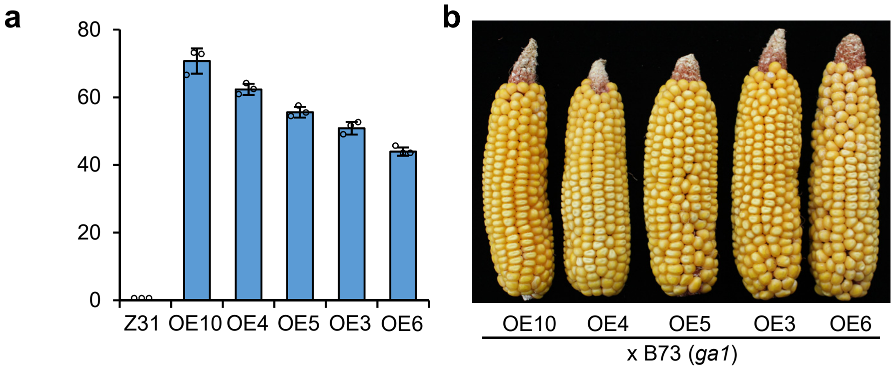

Supplementary Figure 9 Examination of the female function of *ZmGa1P*. (**a**) The relative expression levels of *ZmGa1P* in the silks of five homozygous T_2 transgenic lines (OE10, OE4, OE5, OE3 and OE6) normalized with *ZmGAPDH.* Error bars represent the mean ± SD (n = 3). (**b**) The ears of five *ZmGa1P* OE homozygous T_2 lines (OE10, OE4, OE5, OE3 and OE6) pollinated by B73 (*ga1*) pollen, showing full seed-set.

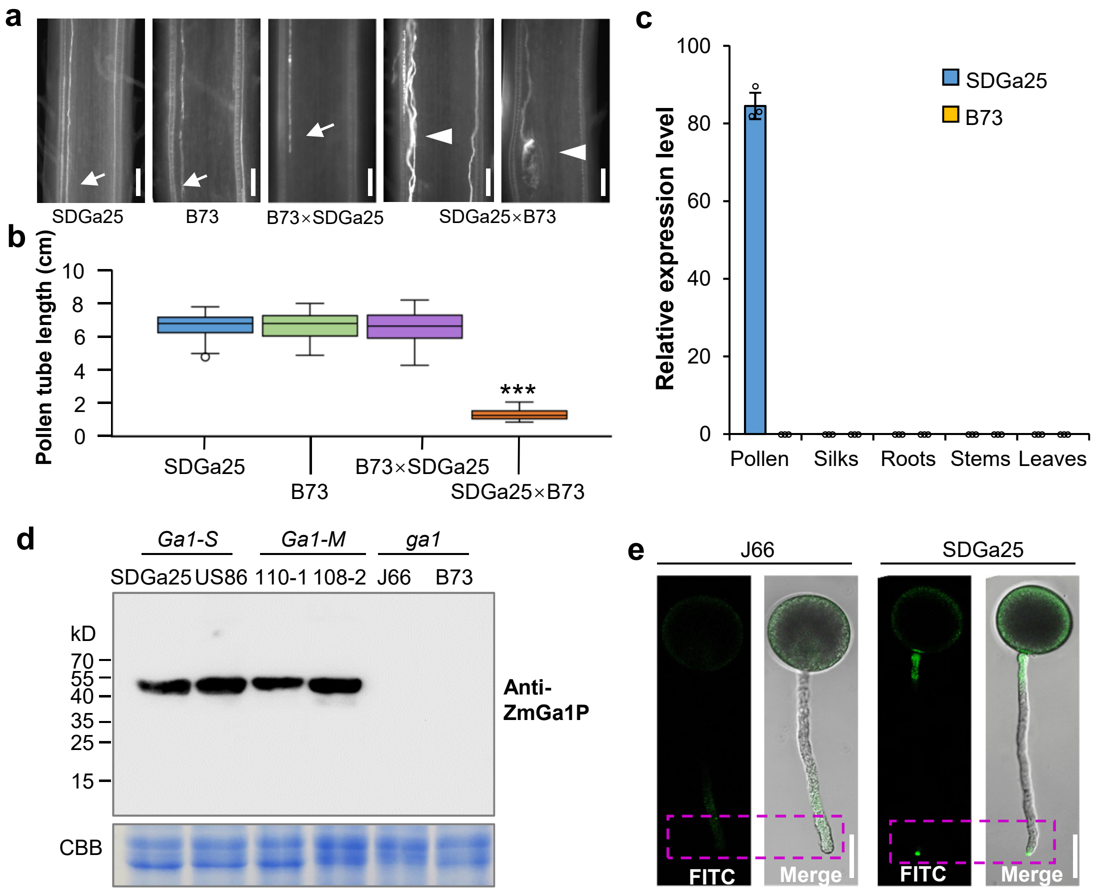

Supplementary Figure 10 *ZmGa1P* is specifically expressed and functions in the pollen tubes. (**a**) Comparison of the *in vivo* pollen tube growth between compatible (SDGa25 selfing, B73 selfing, and B73 x SDGa25) and incompatible (SDGa25 x B73) pollinations. Scale bar = 10 μm. Arrows point to normal pollen tubes and arrowheads indicate short pollen tubes and a clavate pollen tip. (**b**) Box plot of the pollen tube length in the indicated crosses examined in (**a**). Error bars represent the mean ± SD (n = 24). ***P < 0.001 as determined by Student's t-test with Welch's correction (two tailed). (**c**) Spatial and quantitative analysis of *ZmGa1P* expression in SDGa25 (*Ga1-S*) and B73 (*ga1*) normalized with *ZmGAPDH.* Error bars represent the mean ± SD (n = 3). (**d**) Western blotting of secretory proteins from pollen of SDGa25, US86, 110-1, 108-2, J66, and B73 using the anti-ZmGa1P antibody. The amount of protein loaded in each lane was determined by Coomassie brilliant blue staining (CBB). (**e**) Immuno-staining the J66 and SDGa25 pollen tubes using anti-ZmGa1P antibody. The parts circled by magenta dashed lines were enlarged in Fig. 3a. Scale bar = 10 μm. FITC, Fluorescein isothiocyanate.

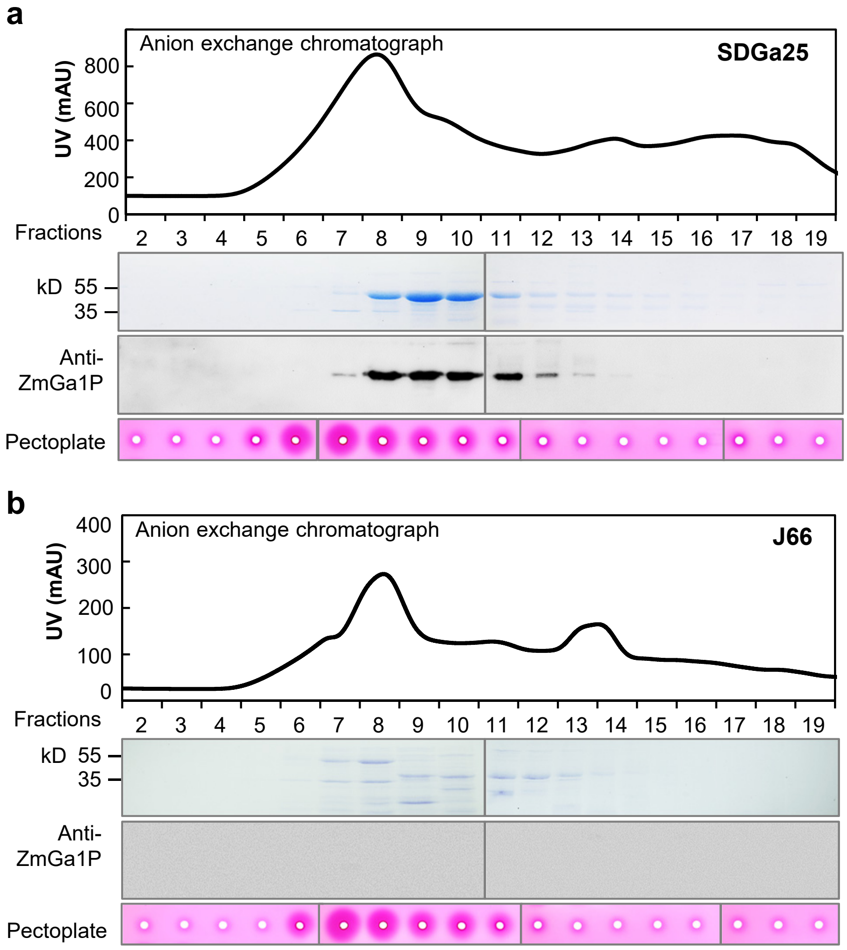

Supplementary Figure 11 Fractionation and pectplate assay of pollen secretory proteins. (**a** and **b**) Fractionation of the total proteins secreted by germinated pollen using successive anion exchange. Equal amount of secretory proteins of fractions 2-19 from SDGa25 (*Ga1-S*, **a**) and J66 (*ga1*, **b**) were separated on SDS-PAGE gel (upper panel) and probed with anti-ZmGa1P antibody (middle panel). Pectoplate assay to show methylesterase activities in the above fractions (bottom panel).

1	MMMSKQMLVL	SLLLVLFELG	SLPTTSCKKV	FFNLWVTNQP	ANATQDAGCA
51	KKDDALSSAD	TIKVWNYIDP	ASQLRPEDGG	YTTISESIAN	IPEDNAKRYL
101	LILKPGVVFR	EKLLLGRSKP	FITIMSEDPM	NPAVIVWNDT	ATTMGKDGKP
151	LGVDGSSTMA	IESDYFVAYN	VVFKNDAPLP	KLGEKKGEAP	ALRVMGTKAT
201	FYNCTIEGGQ	GALYDQTGLH	YFKACAIKGT	IDFIFGSAKS	FYEECKIVSV
251	LKEALVLPLA	PPEQDRSRNP	IEIAPGKSGL	AFKTCTIEGE	GEKIYLGRVG
301	TPVIYSYTNI	GKEIVGIISD	GRDVQTVERG	YYCATFRCYG	PGMSPMVTST
351	LTYVEAIPFL	GIHYISGESW	IPSLPPAEE		

Supplementary Figure 12 LC-MS/MS analysis of ZmGa1P in pollen secretome. The detected peptides are highlighted by red letters.

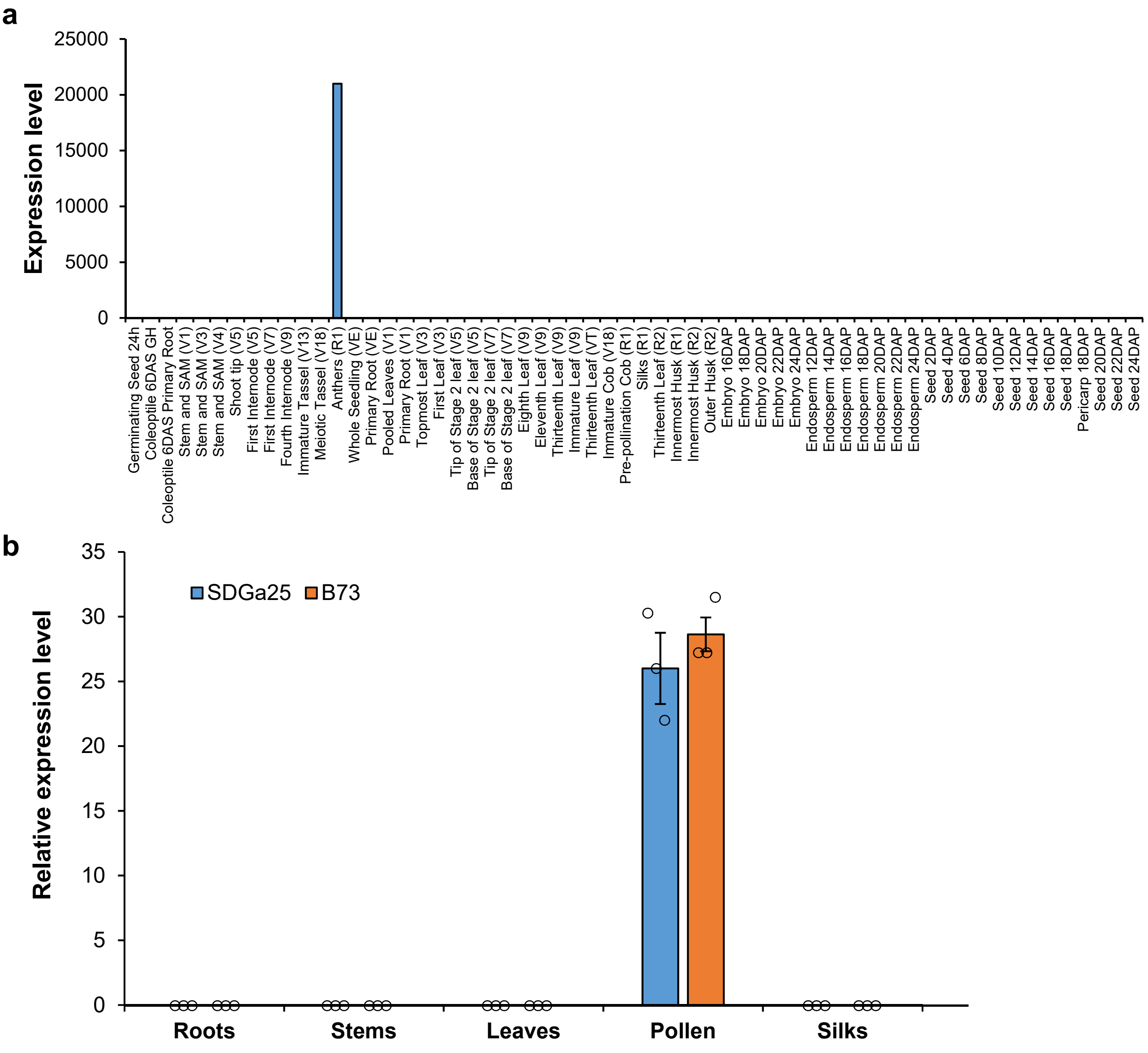

Supplementary Figure 13 *ZmPME10-1* is specifically expressed in pollen. (**a**) Online public RNAseq data[48], showing that *ZmPME10-1* is specially expressed in anthers. (**b**) qPCR to examine *ZmPME10-1* expression in the SDGa25 (*Ga1-S*) and B73 (*ga1*) tissues. *ZmGAPDH* was used an internal control. Error bars represent the mean ± SD (*n* = 3).

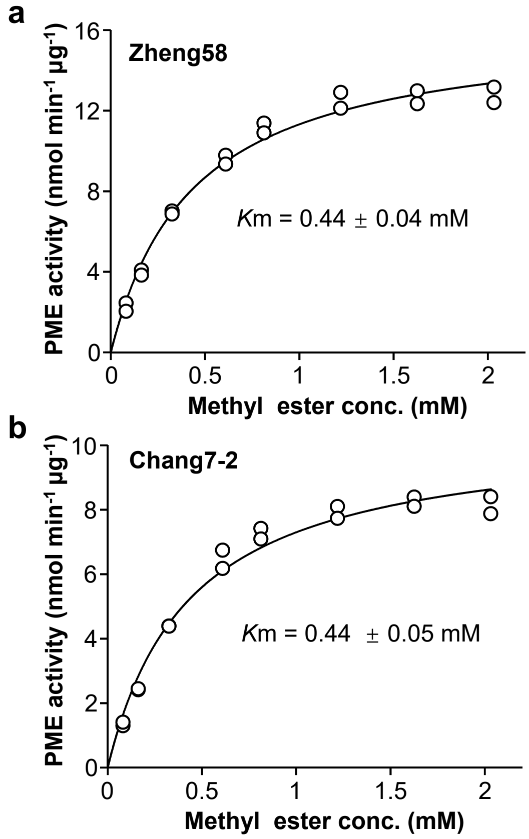

Supplementary Figure 14 Kinetic analysis of PME activity of pollen secretory proteins. (**a** and **b**) Kinetic analysis of PME activity of secretory proteins in fraction 8 of Zheng58 (*ga1*) and Chang7-2 (*ga1*), respectively. The data represent the mean ± SD of three replicates.

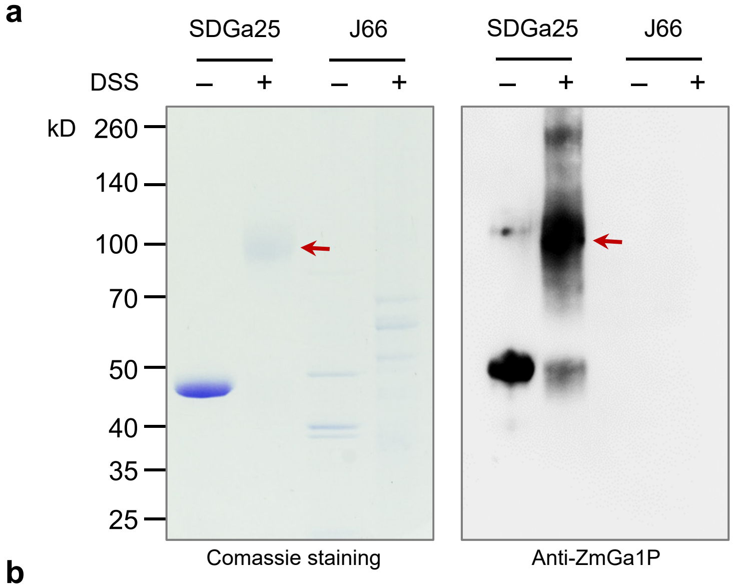

b

Proteins	Locus	PSM	Coverage	Score
ZmGa1P	*Zm00001d048936*	501	58.84	877.19
ZmPME10-1	*Zm00001d026422*	28	14.69	62.85

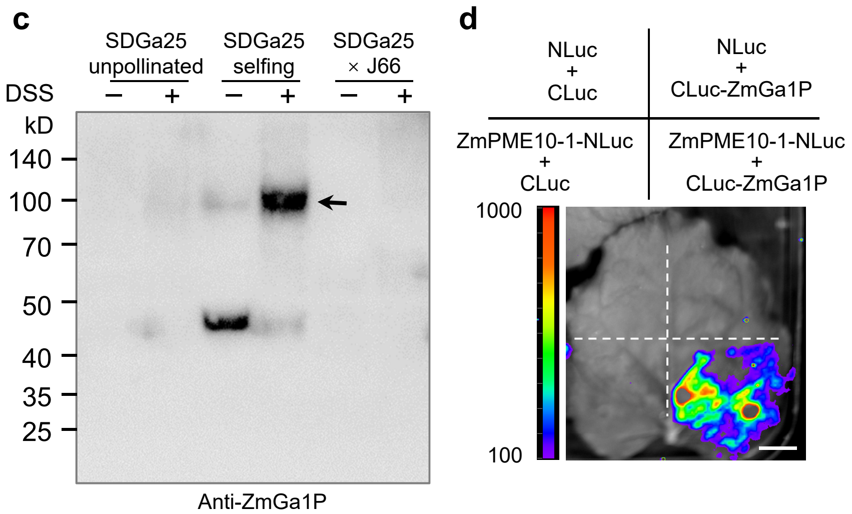

Supplementary Figure 15 ZmGa1P and ZmPME10-1 interact to form a protein complex. (**a**) Western blotting of the pollen secretory proteins crosslinked (+) and not crosslinked (–) by DSS. The left panel is the CBB staining gel containing pollen secretory proteins of SDGa25 (*Ga1-S*) and J66 (*ga1*). The right panel shows the loaded secretory proteins probed by anti-ZmGa1P antibody. The red arrows indicate the 100-kD band. (**b**) LC-MS/MS analysis of the indicated ~100-kD band, identifying ZmGa1P and ZmPME10-1. (**c**) Western blotting of the DSS crosslinked (+) and not crosslinked (–) total proteins extracted from the unpollinated and pollinated silks of SDGa25 using anti-ZmGa1P antibody. The black arrow indicates the ZmGa1P signals at 100-kD position. (**d**) Split-luciferase complementation assay to show the interaction between signal peptide-depleted ZmGa1P and ZmPME10-1 in *N. benthamiana* leaves infiltrated with the construct combinations shown above. Scale bar = 1 cm.

Fig. 2b

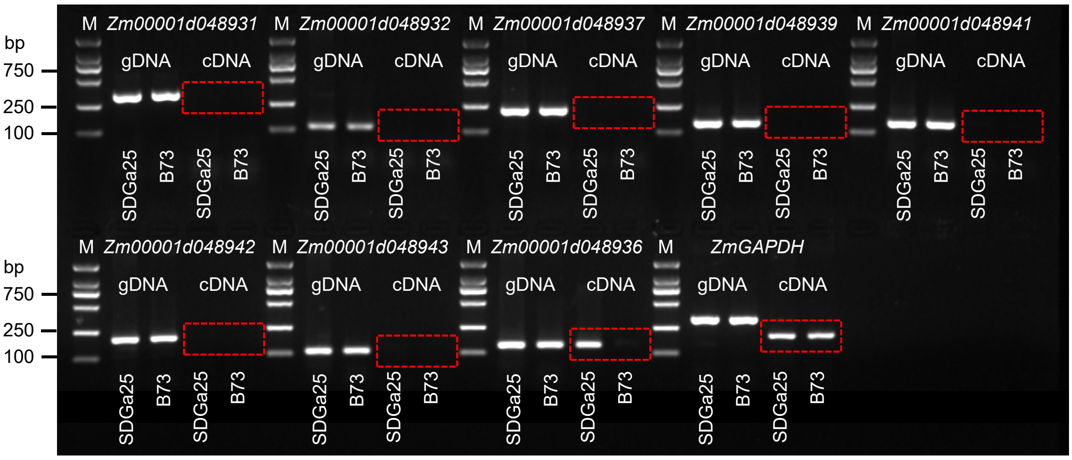

Supplementary Figure 16 Uncropped images of gels shown in Fig. 2.

Supplementary Fig. 2d

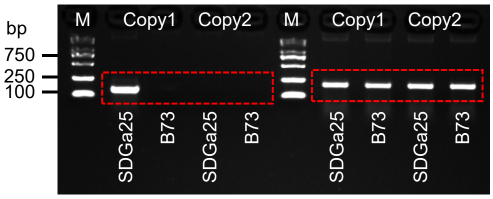

Supplementary Fig. 6

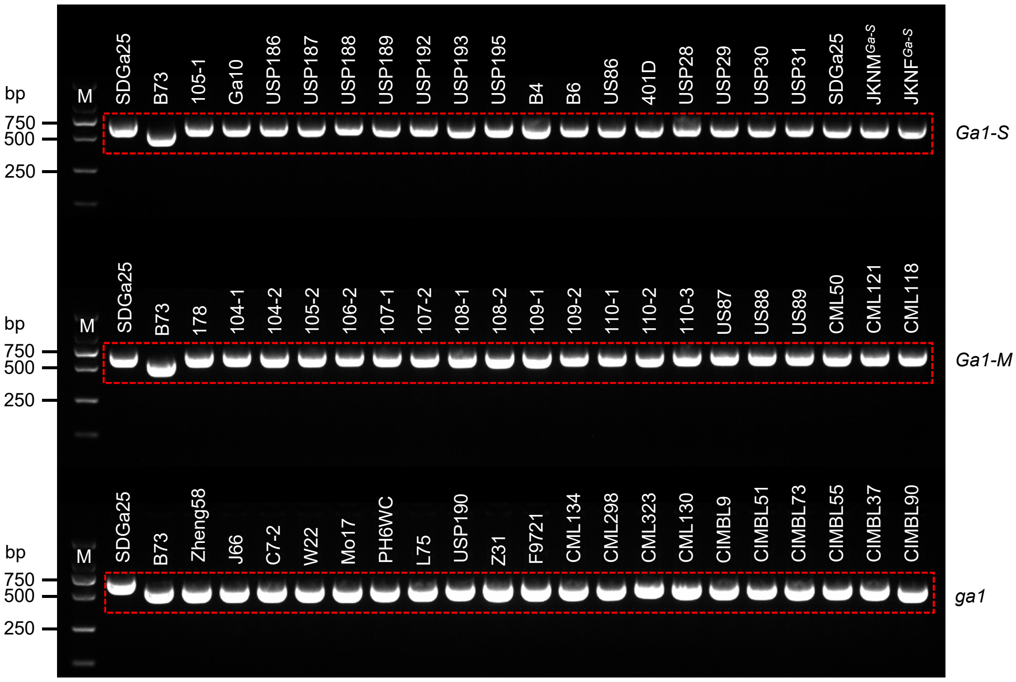

Supplementary Fig. 7b

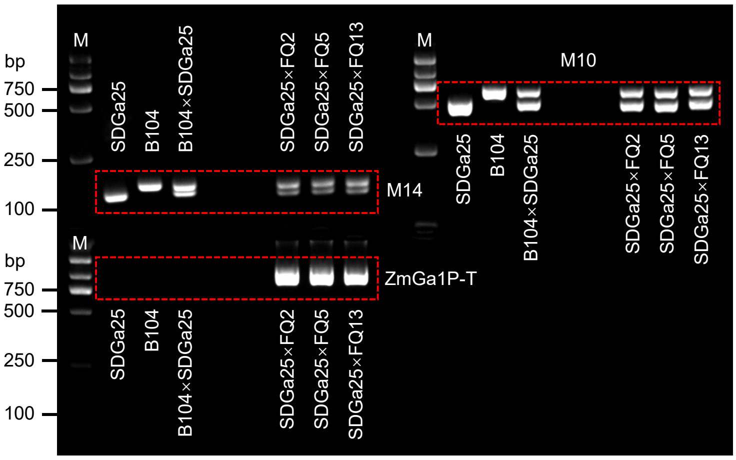

Supplementary Figure 17 Uncropped images of gels shown in Supplementary Fig. 2, 6 and 7.

Supplementary Fig. 8c

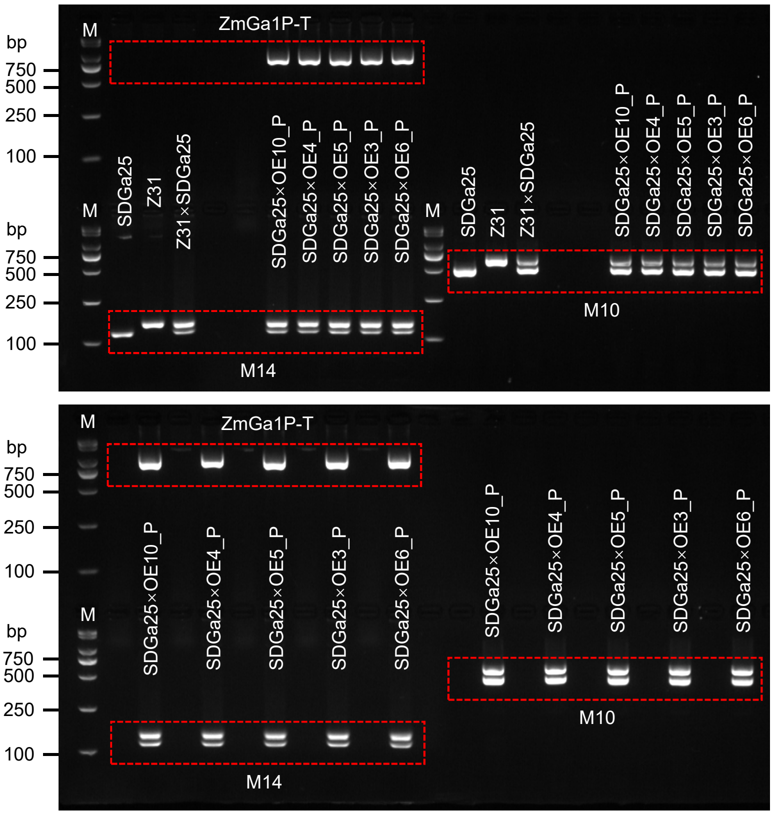

Supplementary Fig. 8d

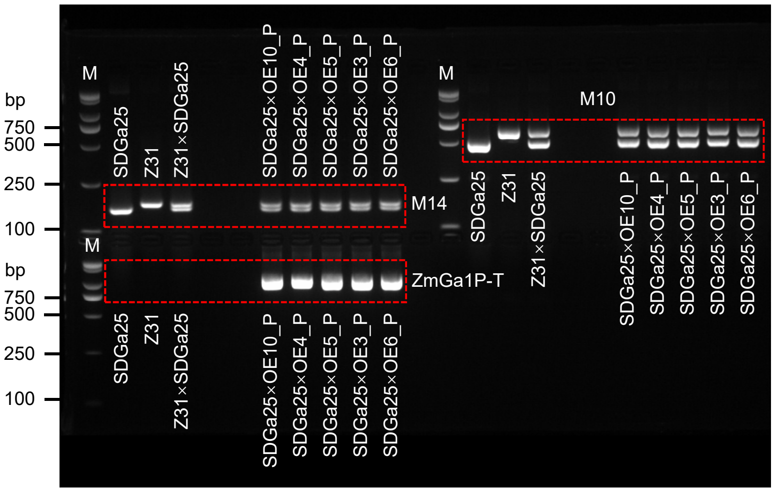

Supplementary Figure 18 Uncropped images of gels shown in Supplementary Fig. 8.

Supplementary Fig. 10d

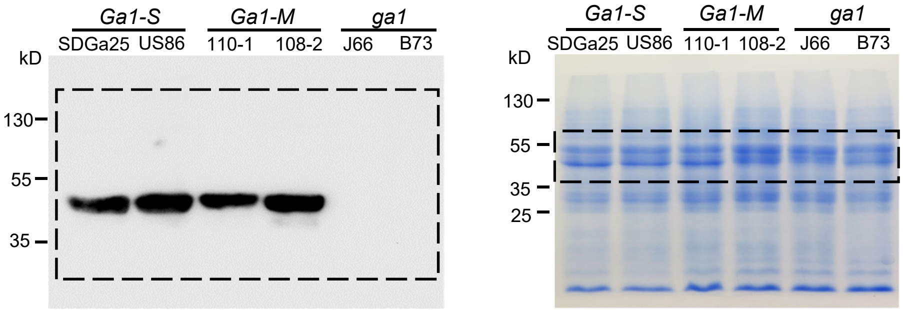

Supplementary Figure 19 Uncropped images of blots and gels shown in Supplementary Fig. 10.

Supplementary Fig. 11a

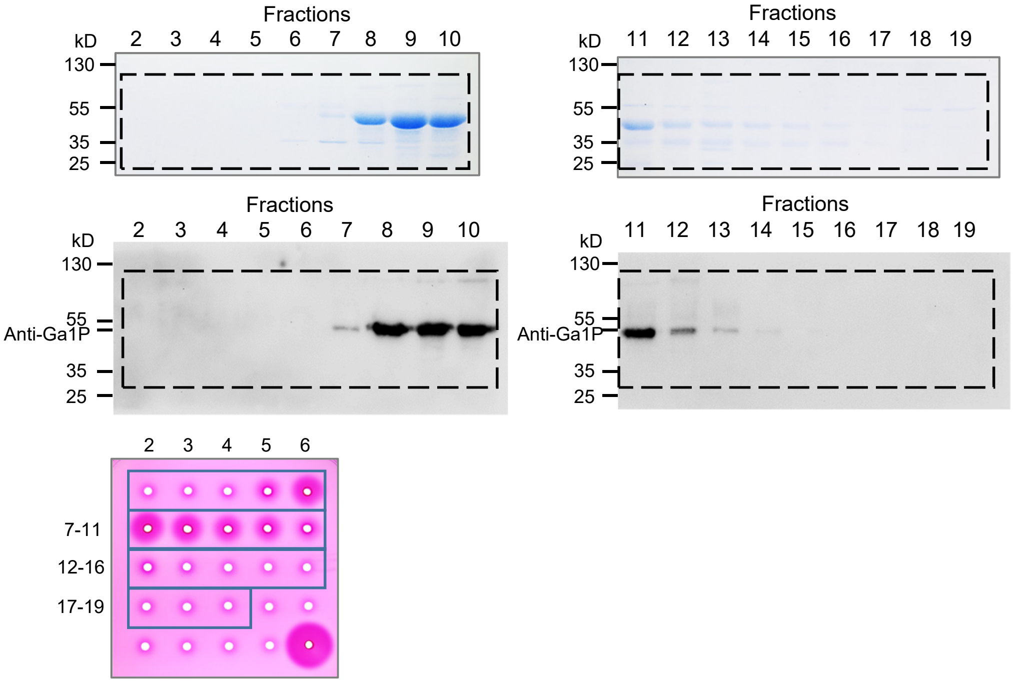

Supplementary Fig. 11b

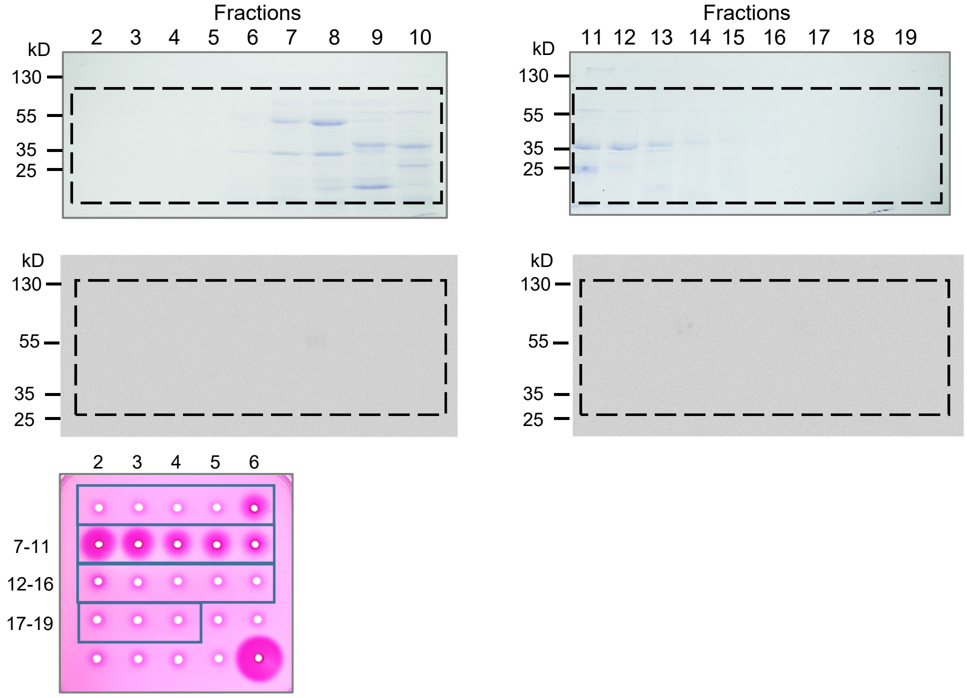

Supplementary Figure 20 Uncropped images of blots and gels shown in Supplementary Fig. 11.

Supplementary Fig. 15a

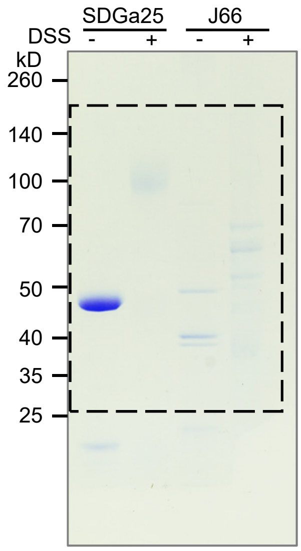

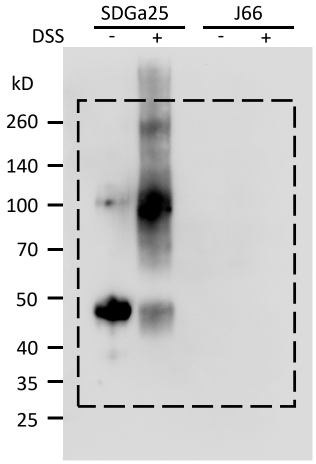

Supplementary Fig. 15c

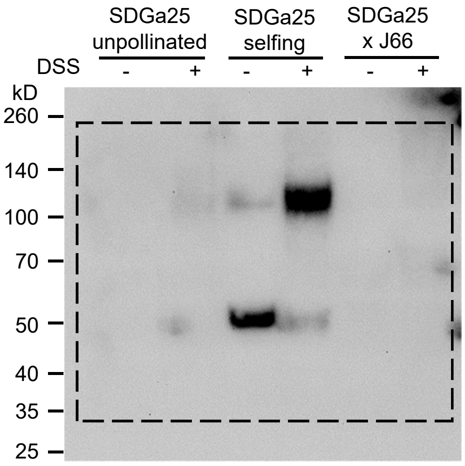

Supplementary Figure 21 Uncropped images of blots and gels shown in Supplementary Fig. 15.

Supplementary Table 1 Genotype and cross-compatibility of the recombinants in the mapping region

Source	Phenotype	No. of recombinants	Marker											
			M14	M13	M33	M44	M53	M61	M1	M4	M16	M89	M10	M11
HP	C	61	Pp	PP	PP	PP	PP	PP	PP	PP	PP	PP	PP	PP
SP	CI	17	Pp	pp	pp	pp	pp	pp	pp	pp	pp	pp	pp	pp
SP	C	14	pp	Pp	Pp	Pp	Pp	Pp	Pp	Pp	Pp	Pp	Pp	Pp
HP	C	43	Pp	Pp	PP	PP	PP	PP	PP	PP	PP	PP	PP	PP
SP	CI	12	Pp	Pp	pp	pp	pp	pp	pp	pp	pp	pp	pp	pp
SP	C	10	pp	pp	Pp	Pp	Pp	Pp	Pp	Pp	Pp	Pp	Pp	Pp
HP	C	21	Pp	Pp	Pp	PP	PP	PP	PP	PP	PP	PP	PP	PP
SP	CI	7	Pp	Pp	Pp	pp	pp	pp	pp	pp	pp	pp	pp	pp
SP	C	5	pp	pp	pp	Pp	Pp	Pp	Pp	Pp	Pp	Pp	Pp	Pp
HP	C	13	Pp	Pp	Pp	Pp	PP	PP	PP	PP	PP	PP	PP	PP
SP	CI	5	Pp	Pp	Pp	Pp	pp	pp	pp	pp	pp	pp	pp	pp
SP	C	3	pp	pp	pp	pp	Pp	Pp	Pp	Pp	Pp	Pp	Pp	Pp
HP	C	7	Pp	Pp	Pp	Pp	Pp	PP	PP	PP	PP	PP	PP	PP
SP	CI	2	Pp	Pp	Pp	Pp	Pp	pp	pp	pp	pp	pp	pp	pp
HP	C	30	PP	PP	PP	PP	PP	PP	PP	PP	PP	PP	PP	Pp
SP	CI	8	pp	pp	pp	pp	pp	pp	pp	pp	pp	pp	pp	Pp
SP	C	11	Pp	Pp	Pp	Pp	Pp	Pp	Pp	Pp	Pp	Pp	Pp	pp
HP	C	27	PP	PP	PP	PP	PP	PP	PP	PP	PP	PP	Pp	Pp
SP	CI	7	pp	pp	pp	pp	pp	pp	pp	pp	pp	pp	Pp	Pp
SP	C	8	Pp	Pp	Pp	Pp	Pp	Pp	Pp	Pp	Pp	Pp	pp	pp
HP	C	12	PP	PP	PP	PP	PP	PP	PP	PP	PP	Pp	Pp	Pp
SP	CI	3	pp	pp	pp	pp	pp	pp	pp	pp	pp	Pp	Pp	Pp
SP	C	3	Pp	Pp	Pp	Pp	Pp	Pp	Pp	Pp	Pp	pp	pp	pp
SP	C	1	Pp	Pp	Pp	Pp	Pp	Pp	Pp	Pp	pp	pp	pp	pp
HP	C	2,000	PP	PP	PP	PP	PP	PP	PP	PP	Pp	Pp	Pp	Pp

C, cross-compatible;
CI, cross-incompatible.
HP, homogeneous mapping population;
SP, BC_1F_1 segregating population;
PP, represents the homozygous genotype identical to SDGa25;
pp, represents the homozygous genotype identical to B73 and J66;
Pp, represents the heterozygous genotype identical to the cross of SDGa25 with B73 or J66.

Supplementary Table 2 The annotated genes and associated SNPs in the mapping region

Gene ID	Chr.	Physical position (AGP_V4)		SNP	Allele	Position[a] (bp)	MAF	*P*-value	Annotated function[b]
		Start site (bp)	End site (bp)						
Zm00001d048931	4	8,536,568	8,538,453	AX-90540670	T/C	8,562,947	0.26	1.03×10^{-14}	Kinesin-like motor protein domain
Zm00001d048932	4	8,540,962	8,542,901	AX-90540670	T/C	8,562,947	0.26	1.03×10^{-14}	TPL-binding domain in jasmonate signalling
Zm00001d048936	4	9,618,917	9,619,330	AX-90540674	C/G	9,619,399	0.09	2.21×10^{-112}	Pectinesterase family protein
Zm00001d048937	4	9,745,227	9,747,622	AX-90857882	T/C	9,743,418	0.20	3.64×10^{-24}	Serine palmitoyltransferase
Zm00001d048939	4	9,751,743	9,752,306	AX-90857912	T/C	9,751,133	0.42	9.69×10^{-28}	Uncharacterized protein
Zm00001d048941	4	10,046,857	10,048,318	AX-90858003	T/C	10,044,364	0.22	1.34×10^{-19}	Fidgetin-like protein 1
Zm00001d048942	4	10,207,633	10,208,412	AX-90540684	A/G	10,216,949	0.22	3.11×10^{-46}	TTF-type zinc finger protein with HAT dimerisation domain
Zm00001d048943	4	10,214,627	10,217,849	AX-90540684	A/G	10,216,949	0.22	3.11×10^{-46}	Thioredoxin-like superfamily protein

[a]Position in base pairs of the lead SNP based on B73 RefGen_V4 sequence. [b]Homology searches against the GenBank of nonredundant protein database and the GenBank conserved domain database were conducted to annotate genes and their conserved domains in the mapped region. Chr., chromosome; MAF, minor allele frequency.

Supplementary Table 3 Putative PMEs identified in the secretome of SDGa25 and J66 pollen

Pollen source	Fractions	Proteins	Gene ID	Score	Coverage	PSM
SDGa25	Q6	ZmPME10-1	Zm00001d026422	547.27	40.37	202
		ZmGa1P	Zm00001d048936	54.75	30.08	25
		ZmPME7-1	Zm00001d022567	53.87	18.02	21
			Zm00001d044585	47.66	28.14	19
SDGa25	Q7	ZmPME10-1	Zm00001d026422	1138.04	51.93	444
		ZmGa1P	Zm00001d048936	413.92	54.09	189
		ZmPME7-1	Zm00001d022567	139.22	29.86	54
			Zm00001d044585	117.64	42.77	46
			Zm00001d014931	11.38	21.99	5
			Zm00001d047251	3.32	2.86	4
SDGa25	Q8	ZmGa1P	Zm00001d048936	902.56	53.83	338
		ZmPME10-1	Zm00001d026422	472.63	33.10	150
		ZmPME7-1	Zm00001d022567	46.46	10.95	15
			Zm00001d044585	14.74	19.46	7
			Zm00001d014931	9.09	20.57	3
SDGa25	Q9	ZmGa1P	Zm00001d048936	304.02	34.04	196
		ZmPME10-1	Zm00001d026422	123.80	21.40	55
		ZmPME7-1	Zm00001d022567	11.60	3.71	3
SDGa25	Q10	ZmGa1P	Zm00001d048936	260.62	37.47	168
		ZmPME10-1	Zm00001d026422	53.21	11.55	18
			Zm00001d014931	6.74	7.80	3
J66	Q6	ZmPME10-1	Zm00001d026422	735.83	47.50	291
		ZmPME7-1	Zm00001d022567	172.52	27.21	64
			Zm00001d044585	76.40	43.93	30
			Zm00001d002124	39.78	12.53	18
J66	Q7	ZmPME10-1	Zm00001d026422	1069.78	51.50	416
		ZmPME7-1	Zm00001d022567	144.34	28.62	59
			Zm00001d044585	108.19	48.27	42
			Zm00001d047251	3.80	2.86	4
J66	Q8	ZmPME10-1	Zm00001d026422	1281.76	50.64	556
		ZmPME7-1	Zm00001d022567	178.25	31.45	78
			Zm00001d002124	62.16	10.15	33
			Zm00001d044585	40.55	25.45	19
			Zm00001d014931	18.13	32.62	8
			Zm00001d047251	3.46	2.86	4
J66	Q9	ZmPME10-1	Zm00001d026422	484.62	33.95	171
		ZmPME7-1	Zm00001d022567	91.41	22.79	37
			Zm00001d044585	33.82	30.35	14
			Zm00001d014931	24.96	39.72	12
J66	Q10	ZmPME10-1	Zm00001d026422	201.20	29.67	77
		ZmPME7-1	Zm00001d022567	30.00	8.30	11
			Zm00001d044585	17.68	11.98	7
			Zm00001d014931	9.80	17.02	4

Supplementary Table 4 Polymorphic markers used for mapping *ZmGa1P*

Primer		Primer sequence (5'-3')	Primer position (bp)	Polymorphism in inbred lines
M14	Forward	GCATGGTTTGCAGGTTCTTG	8102193-8102212	SDGa25 with B73 and J66
	Reverse	CATCTGTGCCTTCACTTCCAG	8102343-8102323	
M13	Forward	TGTGTGACTCCTTGCTCATG	8233435-8233454	SDGa25 with B73 and J66
	Reverse	TCCCTTTCCTCTCTTTTCTTTCT	8233659-8233637	
M33	Forward	CCGTCCTGGTGGGTGGTAC	8337822-8337840	SDGa25 with B73 and J66
	Reverse	GATATAATGGAGGACAGGGAGC	8337920-8337899	
M61	Forward	CGCCTGTGATCTACTCCTACAC	8615352-8615373	SDGa25 with B73 and J66
	Reverse	CCGGCCCTCTATATAGACAGAG	8616398-8616377	
M1	Forward	TTGGCCCATGTCTTCTCA	9958473-9958456	SDGa25 with B73 and J66
	Reverse	ATCTATCGCACAAGCCCTAA	9964029-9964048	
M4	Forward	CCCCTATGATAGAAATGTAGCAC	10215582-10215604	SDGa25 with B73 and J66
	Reverse	CCCACTTGATGTCACCACC	10216278-10216260	
M26	Forward	GTGCATGGCTCTGGTTAC	10470646-10470663	SDGa25 with J66
	Reverse	CCTTGACTACGCCTGAAA	10471347-10471330	
M10	Forward	GGCTTAACTGTAGCCATGCATG	10471346-10471367	SDGa25 with B73
	Reverse	TGAAGAGCTTCGCATGGAGTG	10471986-10471966	
M11	Forward	AGCGATGCGGTTTAGATG	10487937-10487954	SDGa25 with B73 and J66
	Reverse	GCGCCAATAAATGAAGGAT	10488426-10488408	

The primer physical position on chromosome 4 is based on the B73 RefGen_V4 sequence released in maizeGDB.

Supplementary Table 5 SNP markers used for mapping *ZmGa1P*

SNP	SNP position (bp)	Sequences (50 bp upstream and downstream of the SNP)
M44	8445929 [SDGa25/B73/J66]	AGACCAACAGCTTGGATGGCGACTGCATTTTCATCAGCCCCTGCAGCAGC[G/A/A]ATTCATTCTGTGCATGTCCGTATGACGGAGTCGAAGATGATCTTATCTAC
M53	8537889 [SDGa25/B73/J66]	RGTTTGATACTATTAATCTGTGAGTAAAACGTTGCACATTCTACTAWGTA[G/A/A]TAAAAGCTCATGACATAGTGGAACCATCTTGTTGTTTTGCAGAYGCAACA
M16	10265399 [SDGa25/J66]	TTGACTCGCCCGAGGCTAGCCTCTGGTGGGAAGCGCATTTCCGATTCACC[C/T]GAGGGCGTCTCGTGCGATTCTCCGGGAGCTACCCGCCTCGGCCAACCACT
M18	10265470 [SDGa25/B73]	TCCGGGAGCTACCCGCCTCGGCCAACCACTCCGATACAAGAACGTGTGTC[T/C]GTACGTCCACCTGCCCCGCTGAGGGTGCAAAAGTCAACAAAGGACAACAC
M89	10379103 [SDGa25/B73/J66]	TCCCTAATATTTATATAAATGTTGCAGGGTTCTGTGGAGCTTTGATTTGC[T/A/A]TTAGCTCATTTTTTATCTAATCTTCAARATCAATCAGAATCATAGTCAGG

The SNP physical position on chromosome 4 is based on the B73 RefGen_V4 sequence released in maizeGDB. SNPs between SDGa25 and B73 or J66 are indicated by R, W, and Y. R, A/G; W, A/T; Y, C/T.

Supplementary Table 6 Primers used in this study

Primers	Primer sequence (5'-3')	Experimental purpose
Zm00001d048931	Forward GCTGCATTAGCGCCCTTG	Real-time PCR
	Reverse CTGGTTGGTCCAGAACCTCC	
Zm00001d048932	Forward GCATCAAGAAACAGTGGAACAGG	
	Reverse TTAGTGTGCCCTCCACCCTC	
Zm00001d048936 (copy1)	Forward GGTTACACGACCATTAGCGAG	
	Reverse GAATGGCTTGCTTCTACCGAG	
Zm00001d048936 (copy2)	Forward TGTTCCGCGAGAAGCTGTTAC	
	Reverse CCCACATCCACTCACCGAAG	
Zm00001d048937	Forward GTATTTGATGCCCACAAGGTCG	
	Reverse TGCGTTTCCTGCGACACCTC	
Zm00001d048939	Forward GAACGGGTGGGTAGGAGGAG	
	Reverse CAGCAACGACACCGAGCAG	
Zm00001d048941	Forward GGTGGTGGAACGAAGATGTC	
	Reverse CTTGACCCCGGGCTCTAC	
Zm00001d048942	Forward CATTGTTTGCCGGTGGTG	
	Reverse TATTTGGCCTGCGCTTCC	
Zm00001d048943	Forward GCCACGGTGCTGGAGTTC	
	Reverse CGTCAGCGAGCACGAAAC	
Zm00001d026422	Forward GGCCAACATCTTCATGTACGG	
	Reverse CCCATCGACTTGCAGATGAAC	
ZmGa1P-BAC	Forward CAAGGAGGAAGGGAGGAATAAG	BAC scanning
	Reverse GGTGCTCCTAGCATATATGACTC	
ZmGa1P-SDGa25	Forward ATGATGATGAGTAAACAAATGCTCG	*ZmGa1P* Cloning
	Reverse TTATTCTTCAGCGGGTGGTAGG	
ZmGa1P-Del	Forward GATGGAAGCAGTACCATGGC	*ZmGa1P* deletion detection
	Reverse GAGACATCCCAGGCCCGTAAC	
ZmPME10-1-Nluc	Forward GACGACAACGATGGCAACATG	Luciferase complementation
	Reverse TTACCCTTTGGTGAATCCCATG	
CLuc-ZmPME10-1	Forward GACGACAACGATGGCAACATG	
	Reverse TTACCCTTTGGTGAATCCCATG	
Cluc-ZmGa1P	Forward ATGAAAAAGGTCTTTTTCAACTTATGGG	
	Reverse TTCTTCAGCGGGTGGTAGGG	
ZmGa1P-T	Forward ACCTGAAGATGGCGGTTACAC	Transgenic identification
	Reverse CTCAAGCTGCTCTAGCATTCG	
