# Supplementary material for: A PECTIN METHYLESTERASE gene at the maize Ga1 locus confers male function in unilateral cross-incompatibility
Source: Nat Commun. 2018 Sep 10;9:3678. doi: 10.1038/s41467-018-06139-8 (PMC6131150; doi:10.1038/s41467-018-06139-8)
Supplement: Supplementary file 2 — Description of Additional Supplementary Files [file 41467_2018_6139_MOESM2_ESM.pdf]

Legends for Supplementary Data

Supplementary Data 1. The BAC sequence of SDGa25 *ZmGa1P*.

Supplementary Data 2. LC-MS/MS analysis of IEF fraction 6-10 of SDGa25 and J66 pollen secretome.

Supplementary Data 3. LC-MS/MS analysis of the 100 kb DSS-crosslinked proteins in SDGa25 pollen secretome.

Supplementary Data 4. Haplotypes of the 946 inbred lines at *Ga1* locus.
